# Casein kinase 1 controls components of a TORC2 signaling network in budding yeast

**DOI:** 10.1242/jcs.262036

**Published:** 2024-12-20

**Authors:** Rafael Lucena, Akshi Jasani, Steph Anastasia, Douglas Kellogg, Maria Alcaide-Gavilan

**Affiliations:** Department of Molecular, Cell and Developmental Biology, University of California, Santa Cruz, Santa Cruz, CA 95064, USA

**Keywords:** Casein kinase 1, Nutrients, TORC2 signaling, PP2A

## Abstract

Tor kinases play diverse and essential roles in control of nutrient signaling and cell growth. These kinases are assembled into two multiprotein complexes known as TORC1 and TORC2. In budding yeast, TORC2 relays nutrient-dependent signals that strongly influence growth rate and cell size. However, the mechanisms that control TORC2 signaling are poorly understood. Activation of TORC2 requires Mss4, a phosphatidylinositol 4-phosphate 5-kinase that recruits and activates downstream targets of TORC2. Localization of Mss4 to the plasma membrane is thought to be controlled by phosphorylation, and previous work has suggested that yeast homologs of casein kinase 1, Yck1 and Yck2 (referred to here collectively as Yck1/2), Control phosphorylation of Mss4. Here, we generated a new analog-sensitive allele of *YCK2* and used it to test whether Yck1/2 influence localization of Mss4 or signaling in the TORC2 network. We found that Yck1/2 strongly influence Mss4 phosphorylation and localization, as well as influencing regulation of multiple components of the TORC2 network. However, inhibition of Yck1/2 causes mild effects on the best-characterized signaling axis in the TORC2 pathway, suggesting that Yck1/2 might play a larger role in influencing less well-understood aspects of TORC2 signaling.

## Teaser

**Summary:** Yck1/2 kinases regulate Mss4 phosphorylation and localization and affect multiple components of the TORC2 network, influencing lesser-known aspects of this signaling.

## INTRODUCTION

Cell growth is a highly regulated process that requires the orchestration of myriad pathways that are critical for the survival and function of eukaryotic cells. The rate of growth must be tightly linked to nutrient availability to ensure that growth rate is matched to the availability of energy and biosynthetic precursors. Nutrient availability also influences the extent of growth, as the amount of growth required for cell cycle progression is reduced in poor nutrients, which leads to a large reduction in cell size (Fantes and Nurse, 1977; Ferrezuelo et al., 2012; Johnston et al., 1977; Leitao and Kellogg, 2017; Schaechter et al., 1958).

Tor kinases play central roles in the mechanisms that control cell growth. Tor kinases are assembled into two large multiprotein complexes referred to as Tor complex 1 and Tor complex 2 (TORC1 and TORC2, respectively) (Barbet et al., 1996; Heitman et al., 1991; Loewith et al., 2002). TORC1 has been extensively studied and relays nutrient-dependent signals that control metabolism, ribosome biogenesis, autophagy and cell cycle progression (De Virgilio and Loewith, 2006; Moreno-Torres et al., 2015; Wullschleger et al., 2006). Much less is known about TORC2 (Emmerstorfer-Augustin and Thorner, 2023; Riggi et al., 2020). In budding yeast, loss of function of TORC2 network components leads to cell size defects, as well as a failure to modulate growth rate and cell size in response to changes in nutrient availability (Lucena et al., 2018). There is also evidence that TORC2 functions upstream and downstream of membrane tension to maintain homeostasis of cell surface area (Eltschinger and Loewith, 2016; Thorner, 2022)**.**

A key downstream target of TORC2 signaling in budding yeast is a pair of redundant protein kinase paralogs known as Ypk1 and Ypk2 (referred to here collectively as Ypk1/2), which are homologs of mammalian SGK kinases (Casamayor et al., 1999; Fadri et al., 2005; Kamada et al., 2005; Niles et al., 2012). To be activated by TORC2, Ypk1/2 are recruited to the plasma membrane via two redundant protein paralogs named Slm1 and Slm2. A phosphatidylinositol-4-phosphate 5-kinase referred to as Mss4 converts phosphatidylinositol 4-phosphate to phosphatidylinositol (4,5)-bisphosphate, which recruits Slm1 and Slm2 to the plasma membrane (Anand et al., 2012; Yu et al., 2004).

TORC2 signaling is strongly modulated by nutrient availability. For example, TORC2 signaling to Ypk1/2 is high when cells are growing in a rich carbon source like dextrose, and much lower in low-quality carbon sources like glycerol and ethanol (Lucena et al., 2018). The mechanisms by which TORC2 signaling is modulated by carbon source are unknown. A potential clue came from our previous finding that Mss4 undergoes multi-site phosphorylation that is strongly regulated by carbon source. Thus, Mss4 is hyperphosphorylated in rich carbon sources and hypophosphorylated in poor carbon sources (Lucena et al., 2018). Furthermore, there is evidence that increased phosphorylation of Mss4 drives recruitment of Mss4 to the plasma membrane where it can activate TORC2 (Lucena et al., 2018). Taken together, these observations suggest a model in which phosphorylation of Mss4 is an important mechanism by which nutrient availability modulates TORC2 activity (Audhya and Emr, 2003; Lucena et al., 2018). Testing this model will require a better understanding of the signals that control Mss4 phosphorylation, which are largely unknown.

Here, we have investigated the mechanisms that control phosphorylation of Mss4. A previous study has suggested that phosphorylation of Mss4 is dependent upon members of the casein kinase 1γ family (CK1γ), which are encoded in yeast by a pair of redundant paralogs known as Yck1 and Yck2 (referred to here collectively as Yck1/2) (Audhya and Emr, 2003). Cells that lack either paralog are viable, but loss of both is lethal. Yck1/2 are thought to influence diverse cellular processes, such as cell growth, endocytosis, vesicle trafficking, cell cycle progression and glucose sensing (Gadura et al., 2006; Hassaninasab et al., 2019; Moriya and Johnston, 2004; Panek, 1997; Pasula et al., 2010; Robinson et al., 1993; Snowdon and Johnston, 2016; Stalder et al., 2016).

To further investigate the functions of Yck1/2, we generated an analog-sensitive allele of *YCK2* (*yck2-as1*) that can be inhibited with adenine analog inhibitors. We used this new tool to test whether Yck1/2 influence phosphorylation of Mss4 and TORC2 signaling.

## RESULTS

### Generation of an analog-sensitive allele of casein kinase 1

To explore the functions of Yck1/2, we generated an analog-sensitive allele of *YCK2* (*yck2-as1*) that can be rapidly and specifically inhibited *in vivo*. Analog-sensitive kinases are created by mutating a single amino acid in the ATP-binding pocket that confers sensitivity to small-molecule adenine analog inhibitors (Bishop et al., 2000; Lopez et al., 2014). The inhibitor selectively binds to the mutant kinase without affecting other kinases, enabling targeted inactivation of the modified kinase.

**Fig. 1.**
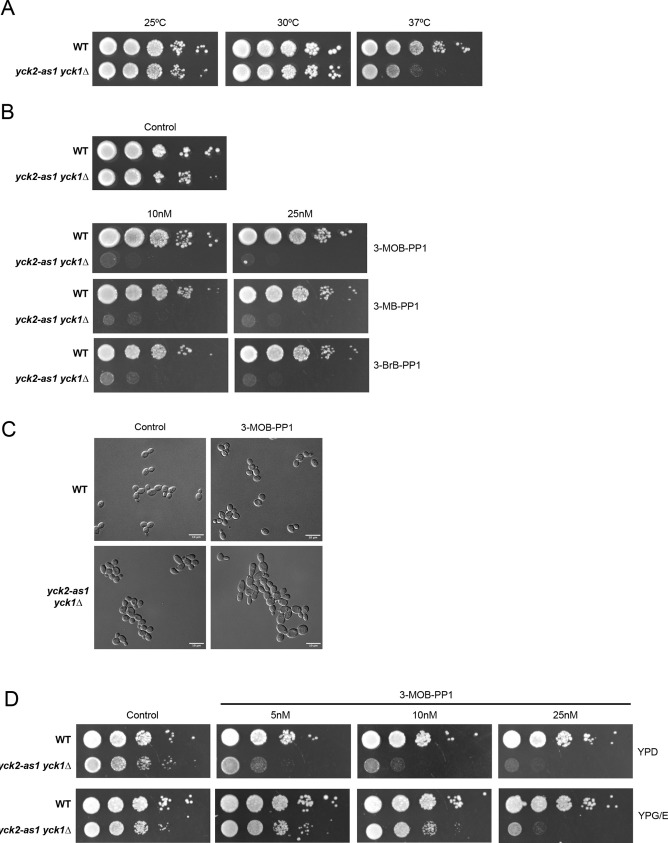
**Characterization of an analog-sensitive allele of *YCK2*.** (A) A series of tenfold dilutions of wild-type (WT) and *yck2-as1 yck1Δ* cells were grown in YPD medium at 25°C, 30°C and 37°C. (B) Series of tenfold dilutions of the indicated strains were grown at 25°C in YPD medium in the absence (control) or presence of different concentrations (10 nM or 25 nM) of the indicated inhibitors (3-MOB-PP1, 3-MB-PP1 and 3-BrB-PP1). (C) Wild-type and *yck2-as1 yck1Δ* cells were grown to log phase in YPD liquid medium at 25°C in the absence or presence of 0.5 µM of the 3-MOB-PP1 inhibitor, and images were taken using a Leica DM8000B microscope. (D) Series of tenfold dilutions of wild-type and *yck2-as1 yck1Δ* cells were grown at 25°C in the absence or presence of different concentrations of 3-MOB-PP1 either in YPD or YEP/GE. Images shown in A,B and D are representative of three independent experiments. Images shown in C are representative of three independent experiments. Scale bar: 10 μm.

Cells carrying the *yck2-as1* allele in a *yck1*Δ background grew slightly slower than wild-type cells at 25°C and 30°C, and substantially slower at 37°C, which indicates that the altered ATP-binding pocket causes reduced activity, as seen for other analog-sensitive alleles (Bishop et al., 2000) (Fig. 1A). The *yck2-as1 yck1*Δ cells showed strong sensitivity to low nanomolar concentrations of analog inhibitors 3-MOB-PP1, 3-BrB-PP1 and 3-MB-PP1 (Fig. 1B). For further experiments, we used 3-MOB-PP1. Addition of 3-MOB-PP1 to *yck2-as1 yck1*Δ cells for 4 h caused cells to become larger, and buds became elongated (Fig. 1C). Similar defects have previously been observed in cells that carry a temperature-sensitive allele of *YCK2* (*yck2-ts yck1*Δ) (Robinson et al., 1993).

Previous work has suggested that Yck1/2 play roles in cells growing in a rich carbon source, such as glucose (Moriya and Johnston, 2004). Here, we found that *yck2-as1 yck1*Δ cells were more resistant to 3-MOB-PP1 in a poor carbon source medium (YP medium with 2% glycerol and 2% ethanol) compared to the same concentration of analog inhibitor in rich carbon source medium, which is consistent with cells having an increased requirement for Yck1/2 activity when in a rich carbon source (Fig. 1D).

### Casein kinase 1 and PP2A^Rts1^ play opposing roles in regulation of Mss4

Previous work has suggested that Yck1/2 regulate Mss4 phosphorylation (Audhya and Emr, 2003). These previous studies utilized a temperature-sensitive allele of *YCK2* (*yck2-ts*) in a *yck1*Δ background and found that inactivation of *yck2-ts* for 30 min at the restrictive temperature caused loss of Mss4 phosphorylation, as assayed via an electrophoretic mobility shift of Mss4 labeled with ^35^S *in vivo*. Previous work has also suggested that hyperphosphorylation of Mss4 is restrained by protein phosphatase 2A (PP2A) associated with the Rts1 regulatory subunit (PP2A^Rts1^) (Lucena et al., 2018). We therefore used the *yck2-as1* allele to evaluate the roles of Yck1/2 and PP2A^Rts1^ in regulation of Mss4. We used western blotting to detect changes in Mss4 electrophoretic mobility caused by phosphorylation (Lucena et al., 2018).

**Fig. 2.**
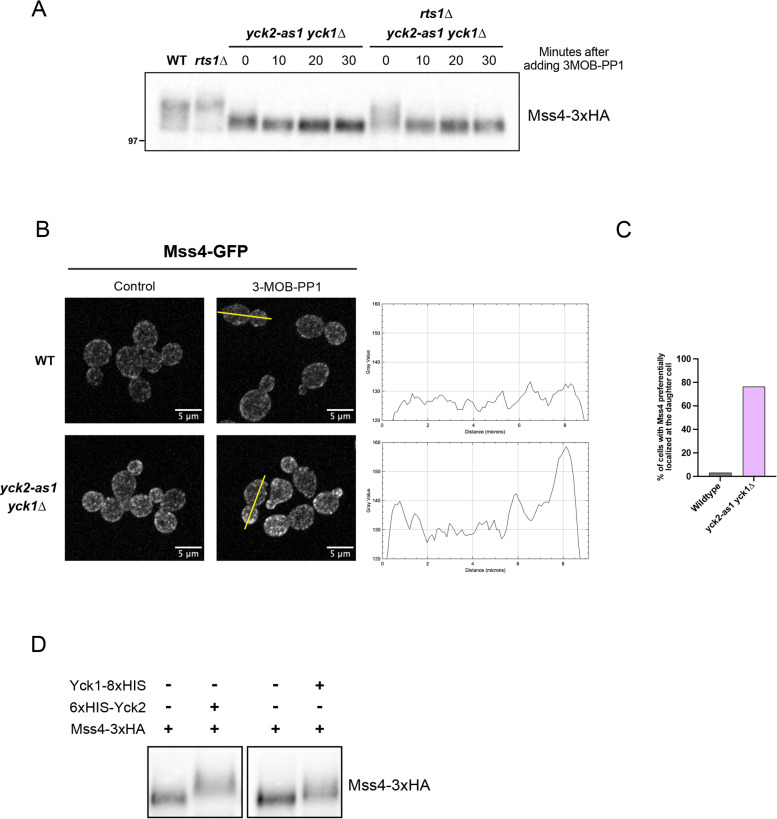
**Yck1/2 are required for normal phosphorylation of Mss4.** (A) Cells of the indicated genotypes were grown in YPD medium to early log phase at 25°C, and 0.5 µM of 3-MOB-PP1 was then added to all *yck2-as1* cells before samples were collected at the indicated time points. Mss4–3×HA was detected by western blotting using an anti-HA antibody. Molecular mass is indicated in kDa. WT, wild type. Blot shown is representative of three independent experiments. (B) Wild-type and *yck2-*as1 *yck1*Δ cells were grown in SD medium with 2% dextrose to early log phase at 25°C and incubated for 30 min with 0.5 µM of 3-MOB-PP1. Cells without addition of 3-MOB-PP1 are shown as a control. Maximal projection of representative cells is shown. Cells were imaged by fluorescence microscopy as described in the Material and Methods. Signal intensity was quantified along a line that bisected the cell, as indicated. (C) Quantification of cells as in B with inhibitor, showing an increase in Mss4–GFP localization in daughter cells (*n*=100). (D) Kinase reactions containing purified Yck1–8×HIS, 6×HIS–Yck2 and Mss4–3×HA in the indicated combinations were initiated by addition of 2 mM ATP and performed for 30 min. Mss4–3×HA was detected using an anti-HA antibody. Blots shown are representative of two independent experiments.

We compared phosphorylation of Mss4 in wild-type, *rts1*Δ, *yck2-as1 yck1*Δ and *yck2-as1 yck1*Δ *rts1*Δ cells before and after addition of analog inhibitor. Mss4 was hyperphosphorylated in *rts1*Δ cells, as previously reported (Fig. 2A, compare lanes 1 and 2) (Lucena et al., 2018). Mss4 phosphorylation was strongly reduced in *yck2-as1 yck1*Δ cells in the absence of inhibitor, consistent with reduced kinase activity of the mutant (Fig. 2A, compare lanes 1 and 3). Addition of 3-MOB-PP1 caused further loss of the phosphorylated forms of Mss4 (Fig. 2A, compare lanes 3 and 4). *rts1*Δ caused an increase in Mss4 phosphorylation in the *yck2-as1 yck1*Δ cells in the absence of inhibitor (Fig. 2A, compare lanes 3 and 7). Addition of 3-MOB-PP1 caused rapid loss of Mss4 phosphorylation in the *yck2-as1 yck1*Δ *rts1*Δ cells. Single deletions of *YCK1* or *YCK2* did not influence Mss4 phosphorylation, which indicates that the two proteins are fully redundant (Fig. S1). Taken together, these data confirm that Yck1/2 strongly influence Mss4 phosphorylation. The fact that loss of Rts1 does not cause all Mss4 to shift to the hyperphosphorylated form and that Mss4 dephosphorylation occurs when Yck1/2 are inhibited in *rts1*Δ cells suggests that multiple phosphatases work on Mss4.

To further test whether Yck1/2 influence Mss4 function, we determined whether normal localization of Mss4 is dependent upon Yck1/2 activity. Wild-type and *yck2-as1 yck1*Δ cells were treated with 3-MOB-PP1 for 30 min and localization of Mss4–GFP was analyzed. (Fig. 2B,C). In wild-type cells, Mss4–GFP was localized primarily to the periphery of both mother cells and daughter buds, as previously reported (Audhya and Emr, 2003; Lucena et al., 2018). In *yck2-as1 yck1*Δ cells, there was increased localization of Mss4–GFP to the periphery, as well as preferential localization of Mss4–GFP to the growing daughter bud. This was surprising because previous work has suggested that localization of Mss4 to the plasma membrane is correlated with phosphorylation of Mss4 (Audhya and Emr, 2003; Lucena et al., 2018). A model that could explain the data is that there are distinct sets of phosphorylation sites on Mss4 that have distinct functions and undergo differential regulation. For example, PP2A^Rts1^ could inhibit phosphorylation of a set of sites that promote localization and activity of Mss4 at the plasma membrane, whereas Yck1/2 influence phosphorylation of a set of sites that inhibits localization of Mss4 to the plasma membrane. Yck1/2 show strong preferential localization to the plasma membrane of the growing daughter bud (Robinson et al., 1999), which could explain why loss of Yck1/2 leads to increased localization of Mss4 to the daughter bud. Furthermore, loss of Yck1/2 leads to increased bud growth, which could be driven in part by increased localization of Mss4 to the growing bud. Overall, the data show that the function and regulation of Mss4 phosphorylation is more complex than previously imagined.

To investigate further, we tested whether Yck1/2 are capable of directly phosphorylating Mss4 *in vitro*. We found that full-length Yck2 purified from bacteria and Yck1 purified from insect cells were both capable of inducing hyperphosphorylation of purified Mss4 *in vitro*, consistent with the possibility that Yck1/2 directly phosphorylate Mss4 (Fig. 2D). However, the data do not rule out models in which Yck1/2 activate another kinase that phosphorylates Mss4 or inhibit a phosphatase that acts on Mss4.

### PP2A influences Yck1/2 phosphorylation *in vivo* and *in vitro*

We found that PP2A^Rts1^ purified from yeast was not able to dephosphorylate Mss4 *in vitro*, even though it was active against a number of other substrates (Fig. S2), which suggests that PP2A^Rts1^ acts indirectly to influence Mss4 phosphorylation. An alternative model is that PP2A^Rts1^ leads to an increase in the activity of Yck1/2. Yck1/2 undergo autophosphorylation (Stalder et al., 2016), which could promote their activity. In this case, if PP2A^Rts1^ opposes autophosphorylation it would be an inhibitor of Yck1/2.

**Fig. 3.**
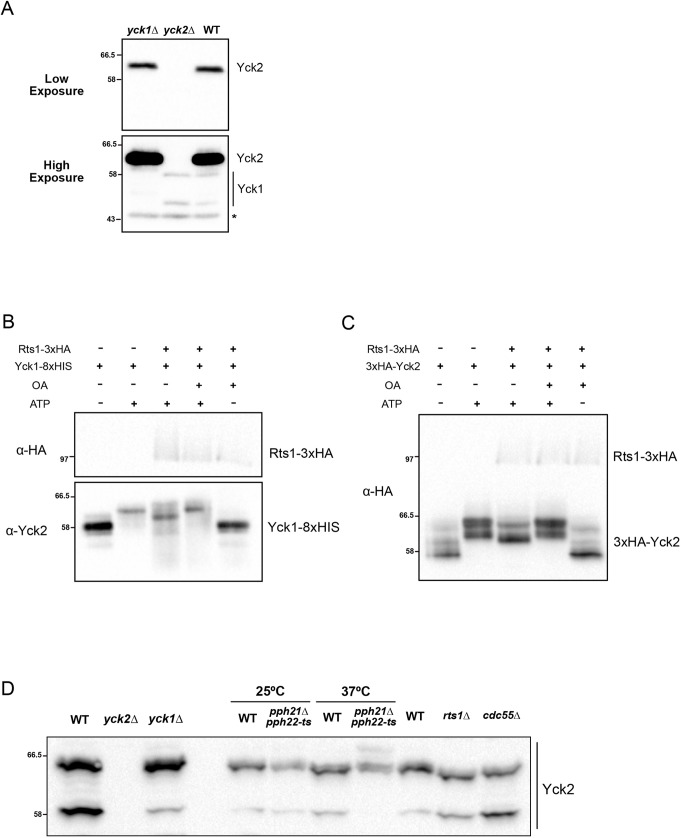
**PP2A influences Yck1/2 phosphorylation.** (A) Extracts were made from strains of the indicated genotypes and used for western blotting using a polyclonal anti-Yck2 antibody. Two different exposures are shown. Background bands are marked with an asterisk. (B,C) *In vitro* assays containing affinity-purified Rts1–3×HA and either Yck1–8×HIS (B) or 3×HA–Yck2 (C) in the indicated combinations were performed for 20 min in the presence or absence of ATP (2 mM) and okadaic acid (OA; 50 µM). Yck1/2 phosphorylation was assayed by western blotting using an anti-HA antibody. (D) Cells of the indicated genotypes were grown to log phase at 25°C in YPD medium. Wild-type and *pph21*Δ *pph22-ts* cells were then shifted to 37°C for 30 min before collecting samples. Phosphorylation of Yck2 was assayed by Phos-tag western blot. Molecular masses are indicated in kDa. WT, wild type. Blots shown in A–D are representative of two independent experiments.

To aid further analysis, we raised an antibody that recognizes Yck2. To validate the antibody, we compared the pattern of bands in wild-type cells to those seen in *yck2*Δ and *yck1*Δ cells to define which bands correspond to Yck2, which showed that the antibody strongly detects Yck2 and weakly detects Yck1 (Fig. 3A).

To test whether PP2A^Rts1^ can oppose autophosphorylation of Yck1/2, we used Yck1–8×HIS purified from insect cells and 3×HA–Yck2 purified from yeast for *in vitro* assays. Both underwent extensive autophosphorylation *in vitro* that could be detected as an electrophoretic mobility shift (Fig. 3B,C). Furthermore, purified PP2A^Rts1^ was able to dephosphorylate Yck1 and Yck2 *in vitro* (Fig. 3B,C).

To test whether PP2A^Rts1^ influences the phosphorylation state of Yck2 *in vivo*, we tested whether inactivation of PP2A catalytic subunits or Rts1 causes hyperphosphorylation of Yck2 *in vivo*. In budding yeast, the PP2A catalytic subunits are encoded by two redundant paralogs that are referred to as *PPH21* and *PPH22*. Cells that lack either paralog are viable, whereas loss of both is nearly lethal (Ronne et al., 1991). To inactivate the catalytic subunits, we used a temperature-sensitive allele of *PPH21* in a *pph22*Δ background (*pph21-172 pph22*Δ) (Evans and Stark, 1997). To obtain better resolution of phosphorylated forms of Yck2, we utilized Phos-Tag gels. We found that Yck2 in log-phase wild-type cells exists in two phosphorylation forms (Fig. 3D). Inactivation of *PPH21* and *PPH22* caused the appearance of a hyperphosphorylated form of Yck2 and loss of a hypophosphorylated form. However, Yck2 did not undergo hyperphosphorylation in *rts1*Δ cells (Fig. 3D). Loss of Cdc55, the other B-type regulatory subunit for PP2A, also had no effect on Yck2 phosphorylation. These observations suggest that forms of PP2A that include Cdc55 or Rts1 cannot be solely responsible for Yck2 dephosphorylation; however, it is possible that PP2A^Rts1^ and PP2A^Cdc55^ work redundantly to mediate PP2A-dependent phosphorylation of Yck2. It is also possible that another form of PP2A is responsible for directly controlling Yck2 phosphorylation. For example, previous studies have shown that PP2A catalytic subunits can also associate with Tap42, which is thought to be regulated by nutrient-dependent signals (Di Como and Arndt, 1996).

### Yck1/2 influence signaling to the Ypk1/2 kinases

**Fig. 4.**
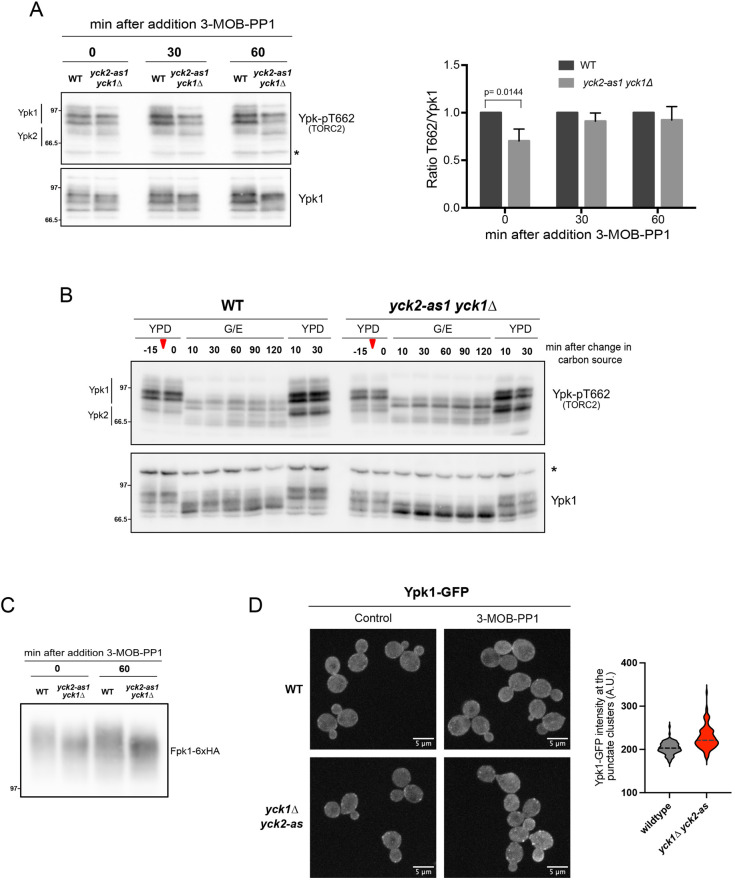
**Yck1/2 regulate components of the TORC2 signaling network.** (A) Wild-type (WT) and *yck1Δ yck2-as1* were grown to early log phase at 25°C and 0.5 µM of 3-MOB-PP1 was the added to both strains before samples were collected at the indicated time points. Ypk-pT662 and anti-Ypk1 antibodies were used for western blotting. Representative blots are shown on the left (asterisk indicates background bands). Quantification of total Ypk-pT662 signal over total Ypk1 protein is shown on the right. Error bars represent the s.d. of the mean of three biological replicates. *P*-value was calculated using a Student's two-sample unpaired *t*-test. (B) Wild-type and *yck2-as1 yck1*Δ cells were grown to early log phase at 25°C, and 0.5 µM of 3-MOB-PP1 was then added to both strains for 15 min before cultures were shifted from YPD to YPG/E medium containing the same amount of inhibitor. After 2 h in YPG/E medium, cells were shifted back to YPD medium (indicated by red arrowheads). Samples were collected at the indicated time points, and Ypk-pT662 and anti-Ypk1 antibodies were used for western blotting analysis (asterisk indicates background bands). Blots shown are representative of four independent experiments. (C) Wild-type and *yck2-as1 yck1Δ* were grown to early log phase at 25°C, and 0.5 µM of 3-MOB-PP1 was then added to both strains before samples were collected at the indicated time points. Fpk1 phosphorylation was assayed by western blotting using an anti-HA antibody. Blot shown is representative of two independent experiments. In A–C, molecular masses are indicated in kDa. (D) Wild-type and *yck2-*as1 *yck1*Δ cells were grown in SD medium containing 2% dextrose to early log phase at 25°C and incubated for 30 min with 0.5 µM of 3-MOB-PP1. Cells without inhibitor treatment are shown as a control. Maximal projections of representative cells are shown on the left. Cells were imaged by fluorescence microscopy as described in the Material and Methods. Right: violin plot showing signal intensity of Ypk1–GFP at the plasma membrane was quantified as described in the Material and Methods (*n*=100; *P*<0.001, Student's two-sample unpaired *t*-test). Median value is represented with a dotted line. A.U., arbitrary units.

Since Yck1/2 are required for normal phosphorylation of Mss4, we next tested whether they influence signaling events in the TORC2 signaling network. To analyze the effects of inhibiting Yck1/2 activity on TORC2 signaling, we utilized a phosphospecific antibody, Ypk-pT662, that detects phosphorylation of Ypk1 at T662, which has previously been identified as a TORC2-dependent phosphorylation site (Niles et al., 2012). The site is also present on Ypk2, so the antibody recognizes phosphorylation of both Ypk proteins at this site. In addition to TORC2-dependent phosphorylation at T662, Ypk1 undergoes multi-site phosphorylation by multiple kinases that cause electrophoretic mobility shifts (Casamayor et al., 1999; Roelants et al., 2002, 2004, 2010; Kamada et al., 2005; Niles and Powers, 2012). Therefore, we also used an antibody that recognizes Ypk1, which allowed us to detect phosphorylation of Ypk1 at sites other than T662 that cause changes in electrophoretic mobility. We found that T662 phosphorylation was modestly reduced in *yck2-as1 yck1*Δ cells in the absence of inhibitor (Fig. 4A). Addition of 3-MOB-PP1 did not cause any further reduction in T662 phosphorylation levels (Fig. 4A). This result is similar to the result obtained for Mss4, where *yck2-as1* caused a loss of phosphorylation of Mss4 in the absence of inhibitor, and addition of inhibitor caused only minimal further effects.

Analysis of Ypk1 phosphorylation via electrophoretic mobility shifts using the anti-Ypk1 antibody showed that *yck2-as1* in the absence of inhibitor caused a change in the phosphorylation state of Ypk1. These differences in the phosphorylation state can be most clearly seen at the 60 min time point (Fig. 4A, bottom panel, compare pattern of bands in wild-type cells versus *yck2-as yck1*Δ cells).

In previous work we found that a shift from rich to poor carbon causes a rapid and dramatic decrease in TORC2-dependent phosphorylation of Ypk1/2, as well a dramatic decrease in overall Ypk1 phosphorylation (Lucena et al., 2018). Therefore, we next tested whether Yck1/2 are required for modulation of TORC2 signaling in response to changes in carbon source. We again saw that *yck2-as1 yck1*Δ cells showed reduced T662 phosphorylation in rich carbon medium before the shift in carbon source. The response of T662 phosphorylation to a shift to poor nutrients appeared to occur normally in *yck2-as yck1*Δ cells (Fig. 4B). However, inhibition of *yck2-as1* caused substantial changes in the electrophoretic mobility of Ypk1, which indicated a loss of phosphorylation at sites other than T662 when cells were shifted to poor carbon medium (Fig. 4B, bottom panel).

Since Yck1/2 are thought to be involved in glucose signaling, we also tested whether TORC2 signaling and Ypk1 respond normally when cells are shifted from a poor carbon source back to glucose-containing medium (Fig. 4B, last two lanes). The response appeared to be normal, again indicating that Yck1/2 are not required for the response of TORC2 signaling to changes in carbon source.

Phosphorylation of Ypk1 that can be detected via electrophoretic mobility shifts is due at least partly to a redundant pair of kinase paralogs called Fpk1 and Fpk2 (also known as Kin82), which play poorly understood roles in regulation of Ypk1/2 (Roelants et al., 2010). To test whether Yck1/2 could control Ypk1 phosphorylation via Fpk1 and Fpk2 (referred to here collectively as Fpk1/2), we tested whether phosphorylation of Fpk1 was dependent upon Yck1/2. We found that Fpk1 phosphorylation that can be detected via electrophoretic mobility shifts was strongly reduced in *yck2-as1 yck1*Δ cells, consistent with the possibility that Yck1/2 influence Ypk1 phosphorylation via Fpk1/2 (Fig. 4C).

To further investigate how Yck1/2 influence Ypk1/2, we analyzed localization of Ypk1–GFP in *yck2-as yck1*Δ cells (Fig. 4D). We found that a reduction in Yck1/2 activity caused increased recruitment of Ypk1–GFP to punctate structures at the cell periphery, which suggests that Yck1/2-dependent phosphorylation events influence localization of Ypk1/2. The identity of the punctate structures is unknown. The fact that reduced activity of Yck1/2 does not cause an increase in TORC2 signaling (Fig. 4A,B) indicates that the structures are unlikely to be sites of TORC2 signaling. The structures do not appear to correlate with the previously reported sites of Fpk1/2 localization at endosomes. Additional experiments will be needed to identify and characterize the sites of increased Ypk1 localization in *yck2-as yck1*Δ cells.

Taken together, the data show that Yck1/2 do not strongly influence TORC2-dependent phosphorylation of Ypk1/2 on T662. Rather, Yck1/2 appear to more strongly influence phosphorylation of a set of poorly understood sites on Ypk1/2 that can be detected via electrophoretic mobility shifts. Ypk1/2 phosphorylation is influenced by the Fpk1/2 kinases, and Yck1/2 appear to influence regulation of Fpk1, which suggests that Yck1/2 could influence Ypk1/2 phosphorylation via regulation of Fpk1/2. Alternatively, there is evidence that Ypk1/2 and Fpk1/2 undergo reciprocal regulation (Roelants et al., 2010), which indicates that another potential model is that loss of Yck1/2 causes an increased ability of Ypk1/2 to phosphorylate Fpk1/2.

### Yck1/2 are required for normal regulation of Rts1 phosphorylation and show genetic interactions with Rts1

**Fig. 5.**
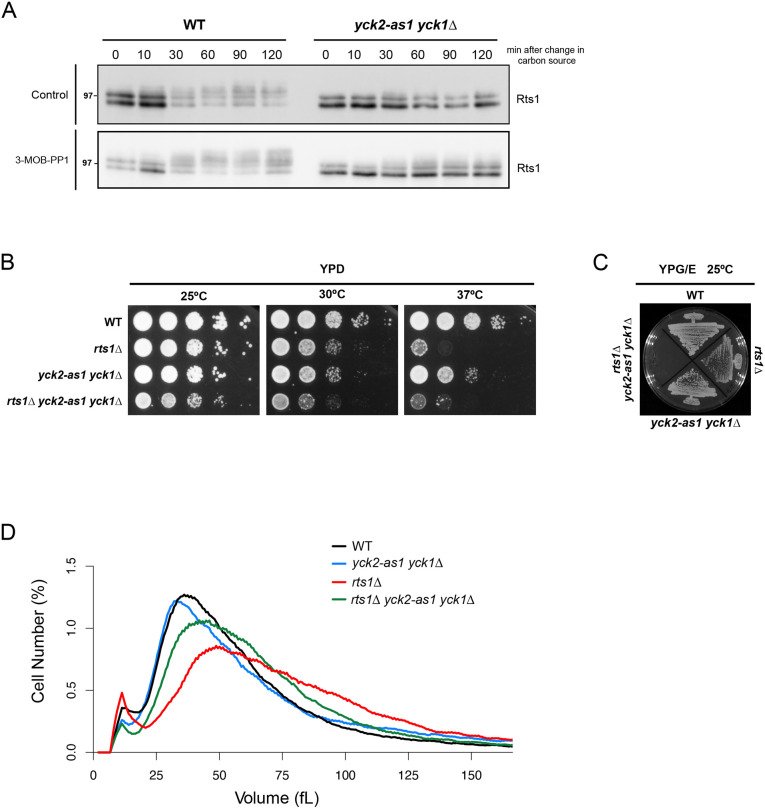
**Yck1/2 and Rts1 show genetic interactions.** (A) Wild-type (WT) and *yck2-as yck1Δ* cells were grown to early log phase at 25°C and cultures were shifted from YPD to YPG/E medium containing 0.5 µM of 3-MOB-PP1 or vehicle (control). Samples were collected at the indicated time points, and Rts1 was analyzed by western blotting using anti-Rts1 antibody. Molecular masses are indicated in kDa. Blots shown are representative of three independent experiments. (B) Series of tenfold dilutions of the indicated strains were grown at different temperatures in YPD medium. Images are representative of three independent experiments. (C) Cells of the indicated genotypes were grown in YPG/E medium at 25°C for 3 days. Image representative of three independent experiments. (D) Cells of the indicated genotypes were grown to log phase at 25°C and cell size distributions were determined using a Coulter Counter. Each plot is the average of three biological replicates. For each biological replicate, three technical replicates were analyzed and averaged.

The TORC2 signaling network influences phosphorylation of Rts1 via poorly understood feedback signals. For example, a shift from rich to poor carbon source causes a dramatic hyperphosphorylation of Rts1 that is dependent upon Ypk1/2, yet Rts1 is also required for normal regulation of Ypk1/2 (Alcaide-Gavilán et al., 2018; Lucena et al., 2018). Although the functions of Rts1 phosphorylation are largely unknown, it provides a readout of signaling events associated with the TORC2 signaling network. To test whether Yck1/2 influence Rts1 phosphorylation, we shifted wild-type control cells and *yck2-as1 yck1*Δ cells from rich to poor carbon sources and inhibited *yck2-as1* with 3-MOB-PP1 at the time of the shift (Fig. 5A). Previous work has shown that all of the electrophoretic mobility shifts of Rts1 that can be detected by SDS -PAGE are due to phosphorylation (Alcaide-Gavilán et al., 2018). Rts1 phosphorylation was already reduced in *yck2-as1 yck1*Δ cells before the shift to the poor carbon source and before addition of inhibitor (Fig. 5A, compare the *t*_0_ time points). Furthermore, hyperphosphorylation of Rts1 failed to occur normally when the *yck2-as1 yck1*Δ cells were shifted to a poor carbon source, both in the presence or absence of analog inhibitor. Rts1 showed a normal response to poor nutrients in both *yck1*Δ and *yck2*Δ cells, again indicating that Yck1 and Yck2 are fully redundant (Fig. S3A). Previous work has shown that inactivation of Ypk1/2 has similar effects on hyperphosphorylation of Rts1 in response to a shift from rich to poor carbon source, consistent with a model in which Yck1/2 influence Ypk1/2 activity via Mss4 (Alcaide-Gavilán et al., 2018).

We next tested for genetic interactions between Yck1/2 and PP2A^Rts1^. We found that *yck2-as1 yck1*Δ slightly reduced the rate of proliferation of *rts1*Δ cells in the absence of inhibitor (Fig. 5B). Furthermore, *yck2-as1 yck1*Δ caused a partial rescue of *rts1*Δ cell size defects in rich medium, but caused a synthetic lethal effect in poor medium (Fig. 5C,D; Fig. S3B). These genetic interactions suggest that Yck1/2 and Rts1 share related functions.

### Membrane localization is required for the ability of Yck1/2 to influence components of the TORC2 network

**Fig. 6.**
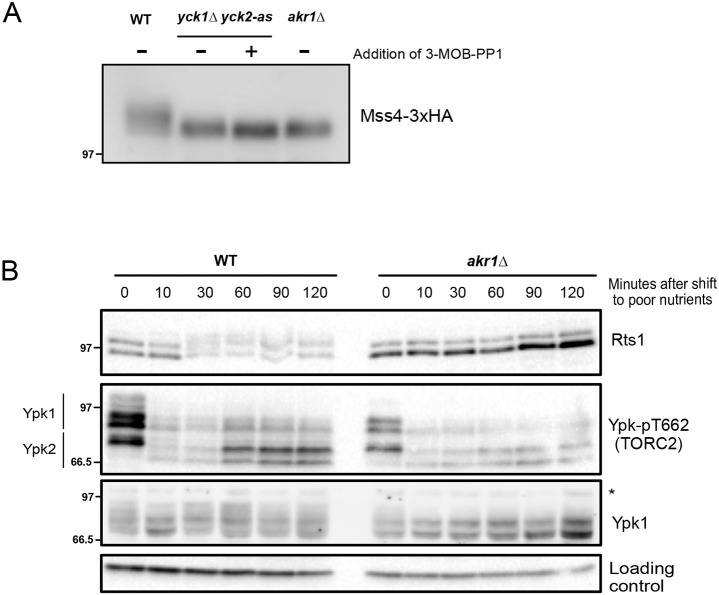
**Membrane localization of Yck1/2 is important for normal regulation of components of the TORC2 signaling network.** (A) Cells of the indicated genotype were grown to early log phase at 25°C in YPD medium and collected and processed for western blotting to analyze Mss4 using an anti-HA antibody. *yck2-as1 yck1*Δ cells were treated with 0.5 µM of 3-MOB-PP1 or vehicle, as indicated, for 10 min before being collected. (B) Wild-type and *akr1*Δ cells were grown to early log phase at 25°C in YPD medium. Cells were washed into YPG/E medium, and samples were collected at the indicated time points. Ypk-pT662 and anti-Ypk1 antibodies were used to analyze Ypk1/2 phosphorylation at the indicated times (asterisk indicates background bands). Rts1 protein was detected using anti-Rts1 antibody. A background band served as loading control. Molecular masses are indicated in kDa. Blots shown in A and B are representative of two independent experiments.

Yck1/2 are anchored in the plasma membrane via a palmitoyl group that is added by a palmitoyl transferase called Akr1 (Babu et al., 2004; Feng and Davis, 2000; Pasula et al., 2010). Cells that lack Akr1 are viable but show defects in cell size and shape that are similar to the effects of inactivating Yck1/2, which indicates that some functions of Yck1/2 are dependent upon membrane localization. The fact that loss Yck1/2 is lethal, whereas loss of Akr1 is not, indicates that not all functions of Yck1/2 are dependent upon membrane localization. We found that *akr1*Δ caused a decrease in Mss4 phosphorylation, as seen for the *yck2-as1 yck1*Δ mutant (Fig. 6A). The *akr1*Δ mutant also showed decreased Ypk1 T662 phosphorylation in rich carbon medium but did not cause a failure in the response of Ypk1 T662 phosphorylation to a shift from rich to poor carbon source (Fig. 6B), as we found for *yck2-as1 yck1*Δ cells. In addition, *akr1*Δ caused defects in hyperphosphorylation of Rts1 in response to a shift to poor carbon source that were similar to the effects of seen in *yck2-as1 yck1*Δ cells. These results suggest that membrane localization of Yck1/2 is required for their effects on components of the TORC2 network. However, since Akr1 also palmitoylates several other proteins, it is possible that loss of membrane localization of several proteins is responsible for the effects of *akr1*Δ.

## DISCUSSION

We developed an analog-sensitive allele of *YCK2* that provides a new tool for analysis of the functions of Yck1/2 in yeast. As a first application, we used the *yck2-as1* allele to analyze how Yck1/2 and PP2A^Rts1^ influence phosphorylation of Mss4. We found that loss of Yck1/2 activity causes a large decrease in phosphorylation of Mss4 that can be detected by electrophoretic mobility shifts. Since Mss4 and Yck1/2 are both localized to the plasma membrane, it is possible that Yck1/2 directly phosphorylate Mss4. We found that purified Yck1 and Yck2 were able to phosphorylate Mss4 *in vitro*, consistent with the possibility that Yck1/2 directly phosphorylate Mss4 *in vivo*.

Mss4 undergoes dephosphorylation even in the absence of Rts1 when there is no Yck1/2 activity (Fig. 2A), which indicates that PP2A^Rts1^ cannot be the only phosphatase that works on Mss4. Loss of Rts1 causes a partial recovery of Mss4 phosphorylation in *yck2-as1 yck1*Δ cells. There are two models that could explain the data. In one model, there are multiple phosphatases that work on the same sites on Mss4. In this case, loss of Rts1 would remove one of the phosphatases, and the other phosphatases do not provide sufficient activity to fully dephosphorylate Mss4. An alternative model is that there are multiple kinases and phosphatases that each work on distinct sets of sites on Mss4. In this case, PP2A^Rts1^ could oppose a kinase that is not Yck1/2, and loss of Rts1 in *yck2-as1 yck1*Δ cells would still lead to hyperphosphorylation of Mss4. It is important to note that detection of phosphorylation events via electrophoretic mobility shifts in standard SDS-PAGE provides limited resolution, as some phosphorylation events cause no shift, and others can cause large or small shifts alone or in combination with other events.

PP2A^Rts1^ purified from yeast was not able to dephosphorylate Mss4 *in vitro* (Fig. S2), which suggests that it acts indirectly. As an alternative we considered a model in which loss of PP2A^Rts1^ leads to an increase in the activity of Yck1/2. We tested this idea and found that PP2A^Rts1^ promotes dephosphorylation of Yck1 and Yck2 *in vitro*. In addition, inactivation of the PP2A catalytic subunit causes hyperphosphorylation of Yck2 *in vivo*. These results are consistent with a model in which PP2A^Rts1^ inhibits Yck1/2. However, loss of Rts1 alone did not cause any effects on Yck2 phosphorylation *in vivo*, which indicates that PP2A^Rts1^ cannot be solely responsible for Yck2 dephosphorylation. It is possible that both PP2A regulatory subunits (Cdc55 and Rts1) work redundantly on Yck2, or that another form of PP2A could be responsible for directly controlling Yck2 phosphorylation.

Phosphorylation of Mss4 is thought to help recruit it to the plasma membrane to promote TORC2 signaling (Audhya et al., 2004; Berchtold and Walther, 2009; Niles et al., 2012). In support of this, we previously found that loss of Rts1 leads to hyperphosphorylation of Mss4, increased recruitment of Mss4 to the plasma membrane and increased TORC2-dependent phosphorylation of Ypk1/2 (Lucena et al., 2018). It was therefore surprising to find that loss of Yck1/2 activity causes a substantial loss of Mss4 phosphorylation but relatively modest effects on TORC2-dependent phosphorylation of Ypk1/2. In addition, inhibition of Yck1/2 caused increased recruitment of Mss4 to the cell periphery, as well as preferential localization of Mss4 to the growing bud. A potential explanation is that a kinase other than Yck1/2 drives phosphorylation-dependent localization of Mss4 to the cell periphery, whereas Yck1/2 influence phosphorylation of a distinct set of sites on Mss4 that play as-yet-undiscovered roles. Yck1/2 are localized to the plasma membrane of the growing bud (Robinson et al., 1999) and we found here that inhibition of Yck1/2 leads to preferential localization of Mss4 to the growing bud. Taken together, these observations suggest that Yck1/2 could inhibit recruitment of Mss4 to the plasma membrane. The data also suggest that Yck1/2 could regulate a function of Mss4 that is not directly tied to the well-characterized role of Mss4 in recruiting TORC2 to the plasma membrane to phosphorylate Ypk1/2.

Previous work has suggested that Yck1/2 play roles in a glucose-sensing pathway (Gadura et al., 2006; Kim et al., 2022; Pasula et al., 2010; Robinson et al., 1992; Snowdon and Johnston, 2016). For example, there is evidence that Yck1/2 are required for normal membrane localization and stability of the Rgt2 glucose sensor, or that Yck1/2 relay a glucose signal to Rgt2. Here, we found that loss of Yck1/2 activity had no effect on the response of TORC2 signaling to changes in carbon source. Thus, in *yck2-as1 yck1*Δ cells, TORC2-dependent phosphorylation of T662 of Ypk1 responded normally to a shift from glucose to a poor carbon source, and to a shift from a poor carbon source to glucose, which indicates that glucose signaling to TORC2 is not dependent upon normal activity of Yck1/2. However, we found that there is a greater requirement for Yck1/2 activity when cells are growing in glucose-containing medium compared to when cells are in poor carbon medium, which is consistent with previous studies that found a role for Yck1/2 in pathways that mediate responses to glucose.

We also tested whether loss of Yck1/2 affects phosphorylation of Ypk1 that can be detected via electrophoretic mobility shifts. Previous studies have suggested that much of the phosphorylation of Ypk1 that can be detected via electrophoretic mobility shifts is dependent upon the Fpk1/2 kinases (Roelants et al., 2010). We found that loss of Yck1/2 caused a loss of Ypk1 phosphorylation that was particularly apparent when cells were shifted from rich to poor carbon sources. We further discovered that loss of Yck1/2 causes a substantial loss of Fpk1 phosphorylation, which suggests that Yck1/2 could influence Ypk1 phosphorylation via activation of Fpk1/2. The functions of Fpk1/2 are poorly understood. Cells that lack Fpk1/2 are viable but have hyperpolarized actin in the growing bud, which suggests that Fpk1/2 control organization of the actin cytoskeleton, potentially via regulation of TORC2 signaling (Rispal et al., 2015). Fpk1/2 are also thought to control phospholipid flippases at the plasma membrane (Roelants et al., 2010). An alternative hypothesis is that Yck1/2 directly phosphorylate Ypk1.

Finally, we tested whether Yck1/2 activity influences phosphorylation of Rts1. In wild-type cells growing in a rich carbon source, Rts1 appeared to be equally distributed between a phosphorylated form and a dephosphorylated form, and a shift from rich to poor carbon source caused further hyperphosphorylation of Rts1. In *yck2-as1 yck1*Δ cells, Rts1 was primarily in the dephosphorylated form and completely failed to undergo hyperphosphorylation when cells were shifted to a poor carbon source. Moreover, we found genetic interactions between Rts1 and Yck1/2 that are consistent with the idea that they share related functions.

The data thus far cannot be explained by a simple model. It is clear that inhibition of Yck1/2 results in a strong loss of phosphorylation of Mss4, Rts1, Ypk1/2 and Fpk1/2, but it does not appear that Yck1/2 can directly phosphorylate all of these proteins, and it is not yet possible to connect all of these events to a single regulatory step controlled by Yck1/2. It is possible that Yck1/2 influences the phosphorylation of each of these TORC2 components via different mechanisms. More work is therefore needed to define the functions and mechanisms of Yck1/2 signaling.

## MATERIALS AND METHODS

### Yeast strains and media

All strains are in the W303 background (leu2-3,112 ura3-1 can1-100 ade2-1 his3-11,15 trp1-1 GAL+ssd1-d2) with the exception of strain LRB1039, which is in an unknown background. Table S1 shows additional genetic features. One-step PCR-based gene replacement was used for making deletions and adding epitope tags at the endogenous locus (Janke et al., 2004; Longtine et al., 1998). Cells were grown in YP medium [1% yeast extract (Life Technologies, 212750), 2% peptone (Thermo Fisher Scientific, 211677), 40 mg/l adenine] supplemented with 2% dextrose (YPD), with 2% galactose (YPGal), or with 2% glycerol and 2% ethanol (YPG/E). For experiments using analog-sensitive alleles, cells were grown in YPD medium without supplemental adenine. For nutrient shifts, cells were grown in YPD medium overnight to log phase. Cells were then washed three times with YPG/E medium and resuspended in YPG/E medium. For microscopy, cells were grown in synthetic minimal medium (0.15% yeast nitrogen base, 0.5% ammonium sulfate) supplemented with the appropriate CSM (MP Biomedicals) and containing 2% glucose (SD) as carbon source.

To create the *yck2-as1* allele, we first amplified the *YCK2* coding sequence along with upstream and downstream non-coding regions and cloned into the BamHI and EcoRI sites of yIPlac211 (laboratory stock; oligonucleotides: 5′-gcgggatccCCGCATATATTCCTAAGTACCTTTTTTTTCAGACAG-3′ and 5′-gcggaattcCTTGATACTCTGTATTTAGTACACAATAACGCCGACG-3′). Site-directed mutagenesis was then used to mutate L149 to G and L90 to I to create pYck2-as-GI. This plasmid was cut with MscI to target integration at Yck2 in a *yck1*Δ background. After selection of transformants, the strain was grown in YPD and plated on 5-fluoroorotic acid to select for recombination events that looped out the plasmid. To identify recombination events that left the mutants in *YCK2*, the isolates were screened for sensitivity to 3-MOB-PP1 and sequenced to verify the presence of the mutations.

The adenine analog inhibitors 3-MOB-PP1, 3-MB-PP1 and 3-BrB-PP1 stock were prepared in 100% DMSO at 10 mM and added to cultures at a final concentration of 5, 10 and 25 nM, as indicated. The analog inhibitors were a gift from Kevan Shokat (University of California, San Francisco).

### Analysis of cell size and cell proliferation assays

Cell cultures were grown overnight to early log phase at 25°C. A 900 ml sample of each culture was fixed with 100 ml of 37% formaldehyde for 30 min and then washed twice with PBS containing 0.04% sodium azide and 0.02% Tween-20. Cell size was measured using a Coulter Counter Z2 (Channelizer Z2, Beckman Coulter) as previously described (Jorgensen et al., 2002). In brief, cells were diluted into 10 ml diluent (Isoton II; Beckman Coulter) and sonicated for 3 s before cell sizing. Each plot is the average of three independent biological replicates in which three independent technical replicates were averaged.

To assay the rate of cell proliferation on plates, cells were grown overnight in the indicated medium at 25°C and adjusted to an OD_600_ of 0.5. Tenfold serial dilutions were spotted onto YPD or YPG/E containing DMSO or different concentrations of 3-MOB-PP1, 3-MB-PP1 and 3-BrB-PP1 and incubated at 25°C, 30°C or 37°C for 3 days.

### Microscopy

To visualize cells in Fig. 1C, wild-type and *yck2-*as1 *yck1*Δ cells were grown in parallel to early log phase in the absence or presence of 0.5 µM of 3-MOB-PP1. Fields of view containing each genotype were imaged using a Leica DM8000B microscope equipped with an objective lens (HCX PL APO 100×/1.40 OIL PH3 CS), a DFC350FX camera and LAS AF software following the instructions of the manufacturer.

Fluorescence images in Figs 2B and 4D were acquired from cells in the exponential growth phase using a Zeiss Axio Observer 7 inverted microscope equipped with Alpha Plan-Apochromat 100×/1.46 Oil DIC lenses coupled with a Spinning Disk Confocal Yokogawa CSU-W1 head. The system was equipped with solid-state excitation lasers [50 mW, 488 nm (GFP)] and a DBP emission filter (525/50) from 3i (Intelligent Imaging Innovations). Device control and image acquisition were performed using SlideBook 6 software. Presented images correspond to maximal projections of 20 stacks of 0.3 µm each.

### Quantification and statistical analysis

Fluorescence intensity measurements were made using open-source ImageJ (Fiji) software (Schneider et al., 2012) and corrected for background. Statistical analyses and the column and violin plots were made using Prism 10.2.1 (GraphPad Software). Significance was defined by a *P*-value equal to or less than 0.05. For all data following a normal distribution, a Student's two-sample unpaired *t*-test was used.

To quantify Mss4–GFP signal in Fig. 2D, a one-pixel line crossing the mother and daughter cell was used. Intensity from a representative cell was plotted using plot profile tool from ImageJ (Fiji). Ypk1–GFP was quantified using an oval section surrounding the puncta around the cell.

### Production of polyclonal Yck2 antibody

An antibody that recognizes Yck2 was generated by immunizing rabbits with a fusion protein expressed in bacteria. A plasmid expressing full-length Yck2 was constructed with the Gateway cloning system. Briefly, a PCR product that includes the full-length open reading frame for Yck2 was cloned into the entry vector pDONR221 (laboratory stock; pVT87). The resulting donor plasmid was used to generate a plasmid that expresses Yck2 fused at its N terminus to 6×His–TEV, using expression vector pDEST17 (6×His, laboratory stock; pVT89). The 6×His–TEV–Yck2 fusion was expressed in BL21 cells (gift from Seth Rubin, University of California, Santa Cruz, USA) and purified via Ni^2+^ affinity chromatography in the presence of 2 M urea using standard procedures (Complete™ His-Tag Purification Resin, Roche 55671500), yielding 10 mg from 4 l of bacterial culture. A milligram of the purified protein was used to immunize a rabbit. Antibodies were raised by Pocono Rabbit Farm (PA, USA) using their standard protocol. The 6×His–Yck2 fusion protein was coupled to Affigel 10 (Bio-Rad, Hercules, CA, USA) to create an affinity column for purification of the antibody.

### Western blotting

To prepare samples for western blotting, 1.6 ml of culture was collected and centrifuged at 20,000 ***g*** for 30 s. The supernatant was removed, and glass beads (250 µl) were added before freezing in liquid nitrogen. To analyze cells shifted from rich to poor nutrients, cultures were grown in YPD overnight at 25° to an OD_600_ of 0.8. After adjusting optical densities to normalize protein loading, cells were washed three times with a large volume of YPG/E medium and then incubated at 25° in YPG/E for the time course. Cells were lysed by bead beating in 140 µl of 1× sample buffer [65 mM Tris-HCl pH 6.8, 3% SDS, 10% glycerol, 50 mM NaF, 100 mM β-glycerophosphate, 5% 2-mercaptoethanol, 2 mM phenylmethylsulfonyl fluoride (PMSF), and Bromophenol Blue]. The PMSF was added immediately before lysis from a 100 mM stock in ethanol. Cells were lysed in a mini-beadbeater-16 (Biospec Products) at top speed for 2 min. The samples were then centrifuged for 15 s at 20,000 ***g***, placed in a boiling water bath for 5 min, and centrifuged for 5 min at 20,000 ***g***. SDS–PAGE was carried out as previously described (Harvey et al., 2005) at a constant current of 20 mA. Proteins were transferred to nitrocellulose using a Trans-Blot Turbo system (Bio-Rad). After transferring to nitrocellulose, blots were blocked for 15 min in PBST (1× PBS, 0.25 M NaCl and 0.1% Tween 20) containing 3% milk except for anti-pT662, which was blocked in TBST (10 mM Tris-HCl pH 7.5, 100 mM NaCl and 0.1% Tween-20) containing 3% milk. Blots were probed with primary antibody overnight at 4°. Proteins tagged with the HA epitope were detected with the 12CA5 anti-HA monoclonal antibody (1:5000; from David Toczyski. University of California, San Francisco, USA). Rabbit anti-phospho-T662 antibody (Ypk-pT662; 1:5000; from Ted Powers University of California, Davis, USA) was used to detect TORC2-dependent phosphorylation of YPK1/2 at a dilution of 1:10,000 in TBST (10 mM Tris-HCl pH 7.5, 100 mM NaCl, and 0.1% Tween-20) containing 3% milk. Total Ypk1 was detected using anti-Ypk1 antibody (Alcaide-Gavilán et al., 2020) at a dilution of 1:10,000. Total Yck1/2 protein was detected using anti-Yck2 antibody at a final concentration of 2 µg/ml. Total Rts1 protein was detecting using anti-Rts1 antibody at a dilution of 1:5000 (Alcaide-Gavilán et al., 2018). All blots were probed with an HRP-conjugated donkey anti-rabbit IgG secondary antibody (catalog number NA934V; GE Healthcare) or HRP-conjugated donkey anti-mouse IgG antibody (catalog number NXA931; GE Healthcare) or HRP-conjugated donkey anti-goat IgG antibody (catalog number sc-2020; Santa Cruz Biotechnology) for 45–90 min at room temperature and 1:5000 dilution. Secondary antibodies were detected via chemiluminescence with Advansta ECL reagents and a Bio-Rad ChemiDoc imaging system.

For Phos-tag western blots, cells were lysed by bead beating into sample buffer without phosphatase inhibitors. After cell lysis, samples were centrifuged for 1 min at 20,000 ***g*** at 4°C and quickly placed in a boiling water bath for 5 min. Samples were loaded into 10% SDS–PAGE gels supplemented with 60 mM Phos-tag and 120 mM MnCl_2_. To prepare Phos-tag gels, the gel mixture was degassed for 1 min prior to the addition of N,N,N′,N′-tetramethylethylenediamine (TEMED, Sigma-Aldrich), and polymerization was allowed to occur for 1–2 h at room temperature followed by overnight at 4°C. Gels were run at 10 mA for 6 h until a 29-kDa marker was at the bottom of the gel. The gel was incubated for 10 min in transfer buffer (Bio-Rad, 10026938) supplemented with 2 mM EDTA, followed by a second incubation without EDTA. Gels were transferred onto nitrocellulose via the Trans-Blot Turbo Transfer System (Bio-Rad).

Quantification of western blot signals was performed using ImageJ (Schneider et al., 2012). Quantification of Ypk-pT662 signal was calculated as the ratio of the phosphospecific signal (total Ypk-pT662 signal) over the total Ypk1 protein signal, with wild-type signal normalized to a value of 1. At least three biological replicates were analyzed and averaged to obtain quantitative information.

### Immunoaffinity purifications

Immunoaffinity purifications using *GAL-3×HA-Yck2* (DK1646) were performed as follows. Cells containing a 3×HA-tagged copy of Yck2 under the control of the *GAL1* promoter were grown overnight at 30°C in YPG/E medium to OD_600_=0.5. Galactose was added to 1%, and cells were incubated at 30°C for 3 h. The cells were pelleted and ground under liquid nitrogen with a mortar and pestle, and 12 g of cell powder was resuspended in 30 ml of extract buffer containing 2 mM PMSF (50 mM HEPES-KOH pH 7.6, 1 M KCl, 1 mM MgCl_2_, 1 mM EGTA, 5% glycerol, 0.25% Tween-20). Crude extracts were stirred for 7–10 min at 4°C to break up cell chunks before centrifugation. All subsequent steps were performed at 4°C. The extract was centrifuged for 5 min at 10,000 ***g***, followed by an additional centrifugation step at 45,000 ***g*** for 45 min. After the final spin, 15 ml of the extract was added to the anti-HA antibody beads. Beads were equilibrated by washing three times in extract buffer before the addition of extract. The clarified extracts were incubated with 650 μg of affinity-purified rabbit polyclonal HA antibody (Alcaide-Gavilán et al., 2020) bound to 500 μl protein A agarose beads (Bio-Rad) for 3 h. The beads were pelleted by centrifugation at 20,000 ***g*** for 5 min, the supernatant was removed, and the remaining extract was incubated with the beads for an additional 1.5 h at 4°C. The beads were pelleted by centrifugation at 20,000 ***g*** for 5 min and washed three times with 15 ml of ice-cold extract buffer without PMSF. After the final wash, the beads were washed twice with ice-cold elution buffer (50 mM HEPES-KOH pH 7.6, 250 mM KCl, 1 mM MgCl_2_, 1 mM EGTA, 5% glycerol, and 0.1% Tween-20). To elute the protein, 250 μl of elution buffer containing 0.5 mg/ml HA dipeptide (synthesized by GenScript) was added to the column and the flow-through fraction was collected. After a 30 min incubation, another aliquot was added. This was repeated for a total of six fractions. Elution fractions two to five were aliquoted in 10 μl volumes and flash frozen on liquid nitrogen.

For the purification of PP2A^Rts1-3×HA^ (DK660) and Mss4-3×HA (DK2326), a similar protocol was followed, except that cells were grown in YPD and 12 g of powder was resuspended in 30 ml of extract buffer (50 mM Tris-HCl pH 7.5, 700 mM NaCl, 150 mM NaF, 150 mM β-glycerophosphate, 1 mM EGTA, 5% glycerol, 0.25% Tween-20, 2 mM PMSF).

### Baculovirus expression and Yck1 purification

To purify Yck1 protein from insect cells, the *YCK1* ORF was amplified (oligonucleotides: 5′-CCGGGATCCATGTCCATGCCCATAGCA-3′ and 5′-CGGCTCGAGTTAGTGGTGGTGGTGGTGGTGGTGGTGGCAACAACCTAATTTTTGGA-3′), digested with BamHI and XhoI and ligated into a modified pFastBac-GST-TEV-MCS-8×HIS vector (renamed to pAJ10) provided by Seth Rubin (University of California, Santa Cruz). The resulting plasmid pAJ12 (pFastBac-GST-TEV-YCK1-8×HIS) was verified by sequencing. pAJ12 was then transformed into competent DH10 *E. coli* cells (gift from Seth Rubin) according to the product protocol to assemble the *YCK1* construct into a bacmid. Bacmid DNA was then isolated and transfected into SF9 insect cells (gift from Seth Rubin) using Fugene transfection reagent. The baculovirus was isolated from the initial transfection and different amounts of the viral titer were used to infect SF9 cells inoculated in 400 ml culture. On day 3 after infection, cells were pelleted at 2300 ***g*** for 15 min at 4°C. The pellet was resuspended in 20 ml lysis buffer [25 mM Tris-HCl pH 8.0, 300 mM NaCl, 1 mM dithiothreitol (DTT), 5% w/v glycerol, 1 mM PMSF, 1× LPC protease inhibitor cocktail (LPC inhibitor cocktail was made by mixing leupeptin, pepstatin and chymostatin, purchased from Sigma, together in DMSO at 1 mg/ml)] in 50 ml falcon tubes and kept on ice for 15 min. The suspension was then sonicated in 4×30 s cycles (Braun-Sonic U sonicator at the lowest power setting), incubating the tubes on ice for 15 s after each sonication. The remaining extract was centrifuged at 16,000 ***g*** for 35 min at 4°C. The protein extract was then loaded onto a 5 ml glutathione–agarose column (Sigma-Aldrich; washed and equilibrated with 15 ml lysis buffer) at ∼20 ml/h. The column was washed with four column volumes of wash buffer 1 (25 mM Tris-HCl pH 8.0, 500 mM NaCl, 1 mM DTT) at ∼30 ml/h. The GST-tagged protein was eluted from the column using elution buffer 1 (40 mM Tris-HCl pH 8.0, 200 mM NaCl, 20 mM glutathione, 1 mM DTT). The elution buffer was loaded onto the column at ∼20 ml/h and ∼750 µl fractions were collected. The protein-containing fractions were identified by Bradford assay and the pooled protein (∼5 ml) was incubated with 50 µg TEV protease (6×HIS-TEV protease was purified from bacteria using standard procedures) to cleave off the GST tag and incubated overnight at 4°C on a roller. On the next day, a 10 µl sample of the digested protein prep was run on a 12.5% polyacrylamide gel and stained with Coomassie Brilliant Blue to confirm complete cleavage of the GST tag. The remaining protein was loaded onto a 1.5 ml Ni-NTA^+^ column (Sigma-Aldrich) at 10 ml/h. The column was washed with wash buffer 2 (25 mM sodium phosphate buffer pH 7.6, 500 mM NaCl, 20 mM imidazole, 5% glycerol, 0.1% Tween-20, 0.5 mM DTT) at 30 ml/h. Yck1–8×HIS was eluted using elution buffer 2 (25 mM sodium phosphate buffer pH 8.0, 200 mM NaCl, 350 mM imidazole, 5% glycerol, 0.025% Tween-20). For elution, 250 µl elution buffer 2 was added to the column each time, the column was incubated at room temperature for 15 mins and the eluted fraction was then collected. Each fraction was tested for presence of protein using a Bradford assay, and the positive protein fractions were pooled together and dialyzed overnight at 4°C using dialysis buffer (50 mM HEPES pH 8.0, 50 mM KCl, 1 mM MgCl_2_, 1 mM DTT, 30% glycerol).

### *In vitro* assays

All *in vitro* assays were performed with 1:10 dilutions of affinity purified protein stocks with dilution buffer (50 mM HEPES-KOH pH 7.6, 2 mM MgCl_2_, 0.05% Tween-20, 1 mM EGTA, 10% glycerol, 1 mM DTT, 20 μg/ml BSA). To demonstrate that PP2A^Rts1^ can oppose phosphorylation of Yck1/2 *in vitro* (Fig. 3B), 2 μl of diluted Rts1–3×HA was incubated with 5 μl of diluted Yck1–8×HIS and 3×HA–Yck2 in the presence or absence of 2 mM ATP and 50 µM okadaic acid (Sigma-Aldrich). 30 μl reactions were carried out in assay buffer for 20 min at 30°C and was terminated by adding 12.5 μl of 4× protein sample buffer (260 mM Tris-HCl, pH 6.8, 12% SDS, 40% glycerol and 20% 2-mercaptoethanol). To demonstrate the ability of Yck1–8×HIS to phosphorylate Mss4–3×HA (Fig. 2D), 1 μl of diluted Yck1–8×HIS was incubated with 5 μl of Mss4–3×HA in a total volume of 30 μl. The reactions were incubated at 30°C for 30 min and terminated by adding 12.5 μl of 4× sample buffer. The samples were boiled at 100°C for 5 min and 20 μl of each reaction was analyzed by western blotting.

## Supplementary Material



10.1242/joces.262036_sup1 Supplementary information
