# Supplementary material for: Casein kinase 1 controls components of a TORC2 signaling network in budding yeast
Source: J Cell Sci. 2024 Dec 20;137(24):jcs262036. doi: 10.1242/jcs.262036 (PMC11795287; doi:10.1242/jcs.262036)
Supplement: Supplementary information [file joces-137-262036-s1.pdf]

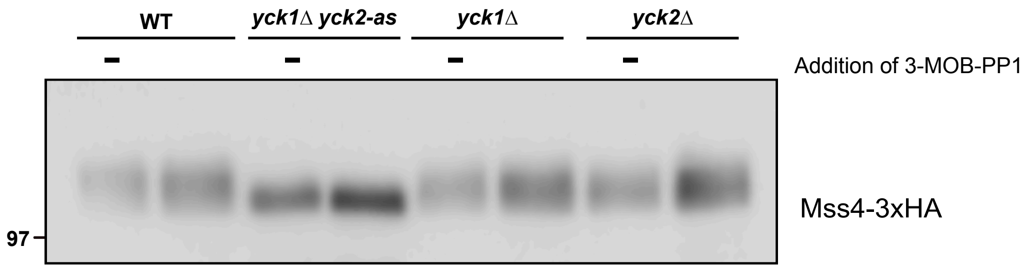

Fig. S1. Cells of the indicated genotype were grown to early log phase at 25ºC in YPD medium and 0.5 µM of 3-MOB-PP1 or vehicle were added for ten minutes. Cells were collected and processed for western blotting to analyze Mss4 using an anti-HA antibody.

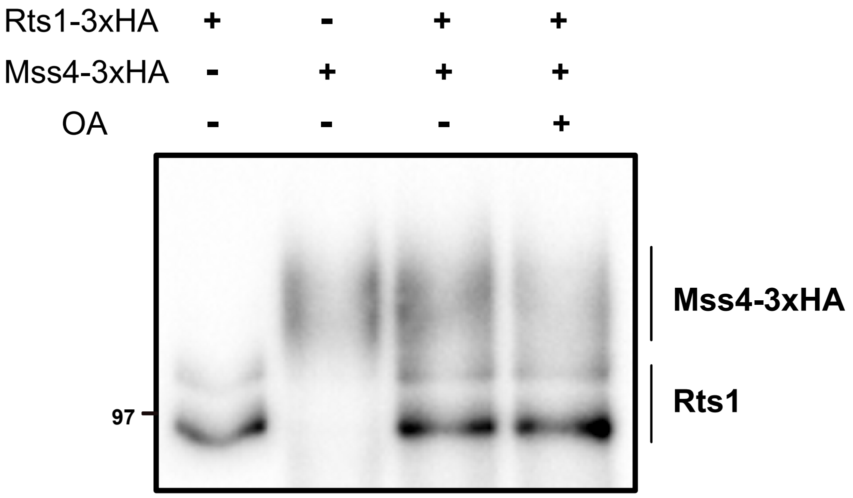

Fig. S2. *In vitro* assays containing affinity purified Rts1–3xHA and Mss4–3xHA in the indicated combinations were performed for 20 min in the presence or absence of ATP (2 mM) and okadaic acid (OA; 50 µM). Mss4 and Rts1 were detected using an anti-HA antibody. Blots shown are representative of two independent experiments.

Journal of Cell Science • Supplementary information

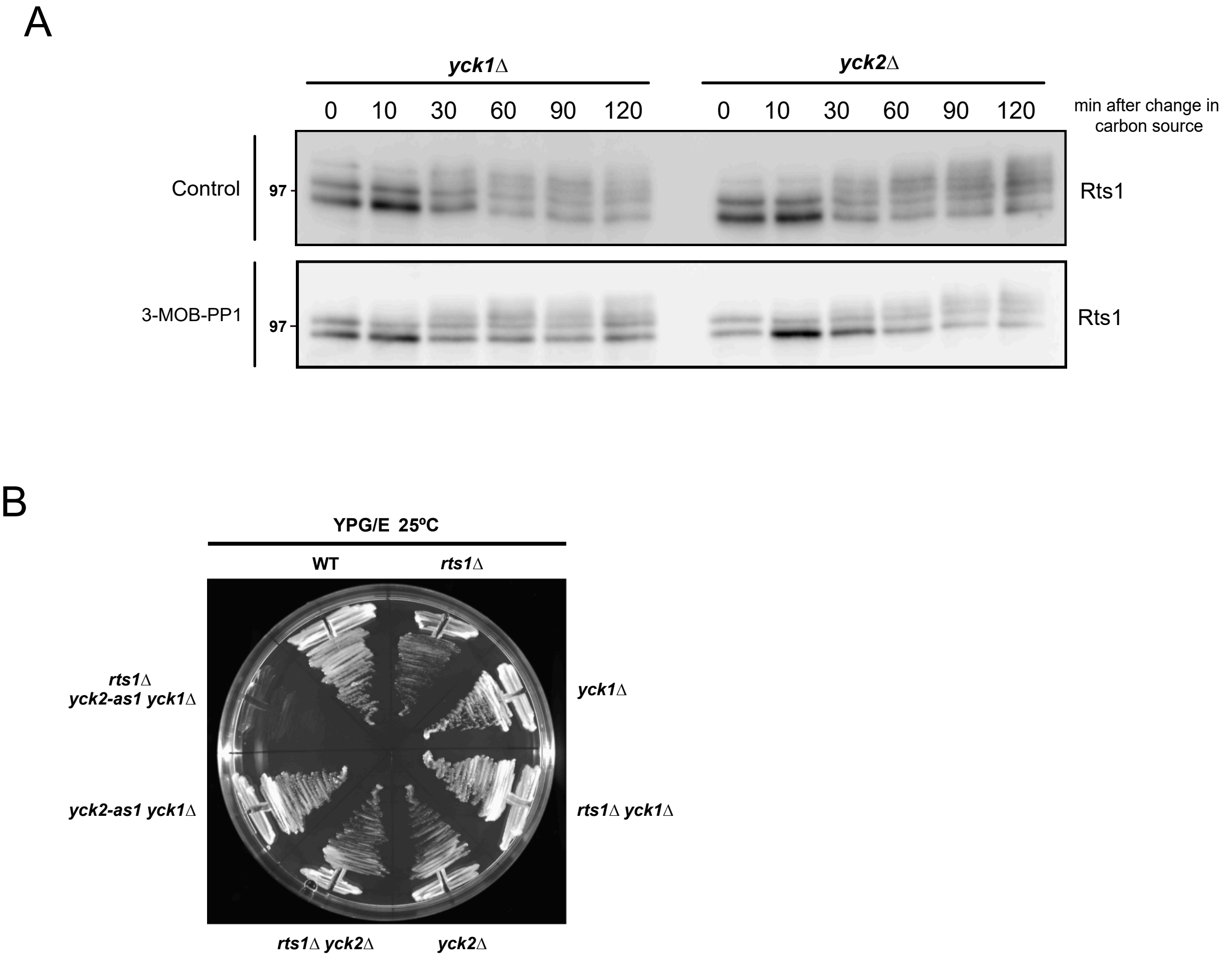

Fig. S3. (A) *yck1Δ* and *yck2*Δ cells were grown to early log phase at 25ºC and cultures were shifted from YPD to YPG/E medium containing 0.5 µM of 3-MOB-PP1 or vehicle. Samples were collected at the indicated time points and Rts1 was analyzed by western blotting using an anti-Rts1 antobody. (B) Cells of the indicated genotypes were grown in YPG/E medium at 25ºC for three days.

Journal of Cell Science • Supplementary information

Table S1. Strains used in this study

Strain	MAT	Genotype	Source
DK186	a	*bar1*	Lab stock
DK647	a	*bar1 rts1Δ::kanMX6*	Lab stock
DK1391	a	*bar1 yck1Δ::KanMX6 yck2-as1*	This study
DK2326	a	*bar1 Mss4-3xHA::HisMX6*	Lab stock
DK2342	a	*bar1 Mss4-3xHA::HisMX6 rts1Δ::kanMX6*	Lab stock
SH341	a	*bar1 pph21Δ1::HIS pph22-172::URA3*	Lab stock
DK2485	a	*bar1 yck1Δ::KanMX6 yck2-as1 Mss4-3xHA::HisMX6*	This study
DK2515	a	*bar1 yck1Δ::KanMX6 yck2-as1 Mss4-3xHA::HisMX6 rts1Δ::Nat*	This study
DK1112	**ɑ**	*bar1 yck1Δ::KanMX6*	This study
LRB1039	a	*his3 leu2 ura3-52 yck2Δ::KanMX*	Lucy Robinson lab
SH650	a	*Bar1 cdc55Δ::kanMX6*	Lab stock
DK660	a	*bar1 Rts1-3xHA:His3MX6*	Lab stock
DK1646	a	*bar1 GAL-3xHA-Yck2::His3MX6*	This study
AL356	a	*bar1 Fpk1-6xHA:His3MX6*	This study
AL358	a	*bar1 Fpk1-6xHA:His3MX6 yck1Δ::KanMX6 yck2-as1*	This study
DK1395	a	*bar1 akr1Δ::TRP*	This study
AL447	a	*bar1 Mss4-GFP::HisMX6*	This study
AL490	a	*bar1 yck1Δ::KanMX6 yck2-as1 Mss4-GFP::HisMX6*	This study
DK3964	a	*bar1 Ypk1-GFP::KanMX6*	This study
AL485	a	*bar1 yck1Δ::KanMX6 yck2-as1 Ypk1-GFP::HisMX6*	This study
AL474	a	*bar1 akr1Δ::TRP Mss4-3xHA::HisMX6*	This study
AL477	a	*bar1 yck1Δ::Nat Mss4-3xHA::HisMX6*	This study
AL479	a	*bar1 yck2Δ::Nat Mss4-3xHA::HisMX6*	This study
