# Supplementary material for: Casein kinase 1 controls components of a TORC2 signaling network in budding yeast
Source: J Cell Sci. 2024 Dec 20;137(24):jcs262036. doi: 10.1242/jcs.262036 (PMC11795287; doi:10.1242/jcs.262036)
Supplement: Supplementary file 1 [file joces-137-262036_review_history.pdf]

Journal of
Cell Science

Casein kinase 1 controls components of a TORC2 signaling network in budding yeast

Rafael Lucena, Akshi Jasani, Steph Anastasia, Douglas Kellogg and Maria Alcaide Gavilan
DOI: 10.1242/jcs.262036

Editor: Megan King

Review timeline
Original submission: 19 February 2024
Editorial decision: 15 April 2024
First revision received: 8 October 2024
Accepted: 16 November 2024

Original submission

First decision letter

MS ID#: JOCES/2024/262036

MS TITLE: Casein kinase 1 controls components of a TORC2 signaling network in budding yeast

AUTHORS: Rafael Lucena, Akshi Jasani, Steph Anastasia, Douglas Kellogg, and Maria Alcaide Gavilan

We have now reached a decision on the above manuscript.

To see the reviewers' reports and a copy of this decision letter, please go to: https://submit-jcs.biologists.org and click on the 'Manuscripts with Decisions' queue in the Author Area. (Corresponding author only has access to reviews.)

As you will see, the reviewers raise a number of substantial criticisms that prevent me from accepting the paper at this stage. One reviewer is more enthusiastic about the contribution to the field, suggesting that a revised version might prove acceptable, if you can address their concerns. The second reviewer, as you will see, is more negative as to impact of the study. If you think that you can deal satisfactorily with the criticisms on revision, I would be pleased to see a revised manuscript. Specifically, I would encourage you to address reproducibility, technical issues raised by the reviewers, and further insights that can be gleaned from subcellular localization studies, as suggested.

Please ensure that you clearly highlight all changes made in the revised manuscript. Please avoid using 'Tracked changes' in Word files as these are lost in PDF conversion.

I should be grateful if you would also provide a point-by-point response detailing how you have dealt with the points raised by the reviewers in the 'Response to Reviewers' box. Please attend to all of the reviewers' comments. If you do not agree with any of their criticisms or suggestions please explain clearly why this is so.

Reviewer 1

Advance summary and potential significance to field

The authors conclude: “The data thus far cannot be explained by a simple model. …

© 2024. Published by The Company of Biologists under the terms of the Creative Commons Attribution License (https://creativecommons.org/licenses/by/4.0/).

More work is therefore needed to define the mechanisms and functions of Yck1/2 signaling within the TORC2 network."

Indeed, it is the opinion of this reviewer that the work in this manuscript is too preliminary for publication in its present form.

Comments for the author

Some general comments:
i/ The authors are proponents of TORC2 regulating cell size control in response to carbon signals but they should not ignore the literature demonstrating that TORC2 functions both downstream and upstream of the biophysical properties of the plasma membrane. In several instances, this literature is cited but not in a proper context.
ii/ Migration of the molecular weight standards should be indicated on all protein gels.
iii/ The authors propose that regulation of Mss4 localization is key to TORC2 control, yet Mss4 localization is never assessed. Indeed, their central tenant that Yck1/2 influences Ypk1/2 activity via Mss4 is not well supported.
iv/ Line 46-47: also cite here Niles et al, 2012; Fadri et al. 2005 doi:10.1091/mbc.e04-07-0564 (and Anand 2012 => PH domain crystal with ligands) regarding Slm1 PH domain v/ Line 75-6: Not all readers are familiar with the ATP analogs used to inhibit AS kinases.
vi/ Fig 2C: why is there much more Mss4-3xHA in lane 4 than in all others??
makes it a bit hard to conclude about a small shift, when loading not equal vii/ Sentences 118-120 are confusing.
viii/ The authors keep saying that "loss of Rts1 causes hyperphosphorylation of Mss4 but does not lead to full hyperphosphorylation" – I don´t understand what they mean. What is full hyperphosphorylation?
ix/ It is unfortunate that the AS kinase appears to be a strong hypomorph. This limits the utility of this approach.
x/ many of the protein shifts are very subtle and the lack of repetitions makes their interpretation tenuous at best.
xi/ Line 201: there is no figure 5D

In summary, this seems like a somewhat random collection of data, the majority of which are negative results. Although alternative hypotheses are presented these generally haven't been followed up. The more positive/striking results have already been published by these authors some years ago (10.1016/j.cub.2017.11.069; 10.1534/genetics.118.301296).
We cannot recommend publication of this manuscript.

Reviewer 2

Advance summary and potential significance to field

This paper examines the role of the budding yeast casein kinase I proteins Yck1 and Yck2 in regulating activation of TORC2-Akt signaling. The authors test a proposed pathway that involves Yck1/2 phosphorylating PI(4,5)P2 kinase Mss4, leading to recruitment of Slm1/2 proteins and TORC2-Akt activation. Yck1 and Yck2 are functionally redundant so that yck1 and yck2 mutants are viable, but the double mutant yck1 yck2 is lethal. Previous work from another group showed that conditional inactivation of yck1Δ yck2-ts mutant prevents phosphorylation of Mss4, leading to this proposed pathway. In the current study, the authors generate an analog-sensitive yck2-as mutant with the goal of testing the pathway more precisely. The yck2-as mutation is a constitutive partial loss-of-function, so combined with yck1Δ provides a partial loss of Yck1/2 function. In this mutant, the authors test phosphorylation of various proteins in the pathway using western blot of whole cell extracts, combined with several in vitro assays using purified proteins. Their results provide a number of new insights for the pathway including: Yck1 might directly phosphorylate Mss4, Yck1/2 activity impacts TORC2 phosphorylation, Yck1/2 and PP2A phosphatase regulate each other's phosphorylation (though probably indirectly), and combined mutations in this pathway impact cell size and viability. The experiments are generally performed well, and the data interpreted appropriately. Each experiment in set up to test a simple prediction for the proposed linear

© 2024. Published by The Company of Biologists under the terms of the Creative Commons Attribution License (https://creativecommons.org/licenses/by/4.0/).

pathway. The results will be helpful for researchers in the yeast cell growth field and are likely to help TORC2-Akt researchers in other experimental systems. The limitation of the study is that the results do not clarify aspects of the pathway. This signaling network clearly involves extensive feedback and likely has other components that are currently unknown. The result is that each finding does not fit into a larger conclusion. The authors are not to blame for the complex network, but it does limit the larger impact of the work. Most of the results lack an underlying mechanism and only tell us that mutant X somehow alters the phosphorylation of protein Y. The ability of the field to understand a complex signaling network requires somebody to test each of these interactions, as the authors have done here. Therefore, the work is important for the field to know, but immediate impact and conclusions are somewhat limited.

Comments for the author

Specific comments:
1. It would be helpful for the authors to test the impact of 3-MOB-PP1 drug on yck1Δ cells, at least for a couple of their assays/results. They need to control for effects of the drug that are independent of the yck2-as1 mutation.

2. In Figure 2C, the loading does not appear equal for the final 2 lanes in the gel. It was hard for me to assess the gel shift for Mss4-3xHA given the difference in intensity between the bands.

3. In Figure 4A, the authors conclude that prolonged incubation with inhibitor alters Ypk1 phosphorylation pattern. I do not see any effect supporting the conclusion.

4. Minor: on line 170, the text states Ypk1/2 but I think they mean Yck1/2.

5. For Figure 5A, it would be helpful to see this experiment performed without inhibitor. Given the minimal effect of inhibitor addition in most experiments, I was confused why it was included here.

6. Based on the model and background information, I am confused why loss of membrane localization does not impact Ypk-pY662 levels in Figure 6. Is it known where Ypk proteins localize in akr1Δ cells shifted to poor carbon? If not, the authors could perform this experiment to clarify their results in Figure 6.
7. Line 242: The text states “these results are consistent with a model in which PP2A dephosphorylates and activates Ypk1/2.” I think they mean “inactivates”? Isn’t the idea that phosphorylation activates Mss4?

8. It would be helpful to know where Mss4 localizes in yck1/2 mutants, based on changes identified in Mss4 phosphorylation.

First revision

Author response to reviewers' comments

Reviewer 1 Advance Summary and Potential Significance to Field...

The authors conclude: “The data thus far cannot be explained by a simple model. … More work is therefore needed to define the mechanisms and functions of Yck1/2 signaling within the TORC2 network.”

Indeed, it is the opinion of this reviewer that the work in this manuscript is too preliminary for publication in its present form.

Reviewer 1 Comments for the Author...

Some general comments:

© 2024. Published by The Company of Biologists under the terms of the Creative Commons Attribution License (https://creativecommons.org/licenses/by/4.0/).

i/ The authors are proponents of TORC2 regulating cell size control in response to carbon signals but they should not ignore the literature demonstrating that TORC2 functions both downstream and upstream of the biophysical properties of the plasma membrane. In several instances, this literature is cited but not in a proper context.
We have edited the citations and added new text to highlight a role for TORC2 in the biophysical properties of the plasma membrane.

ii/ Migration of the molecular weight standards should be indicated on all protein gels.
The figures have been revised to include molecular weight markers on all protein gels in the figures.

iii/ The authors propose that regulation of Mss4 localization is key to TORC2 control, yet Mss4 localization is never assessed. Indeed, their central tenant that Yck1/2 influences Ypk1/2 activity via Mss4 is not well supported.
We carried out new experiments to compare the localization of Mss4 in wild type and *yck-as1* cells, which are shown in a new Figure 2B. The data indicate that loss of Yck1/2 causes increased localization of Mss4 to the plasma membrane, as well as preferential localization of Mss4 to the growing bud. These data indicate that the roles of Mss4 phosphorylation are more complex than previously imagined. Overall, these and other data in the manuscript indicate that Yck1/2 influence multiple components of the TORC2 network, but do not strongly influence phosphorylation of Ypk1/2 by TORC2, which is the best understood signaling axis in the TORC2 network. Inhibition of Yck1/2 therefore helps reveal aspects of the TORC2 network that are much less well-understood, and our hope is that these new data will provide important clues that lead to new insight into the TORC2 network. We have modified the abstract and text to incorporate discussion of these new data.

iv/ Line 46-47: also cite here Niles et al, 2012; Fadri et al. 2005 doi:10.1091/mbc.e04-07-0564 (and Anand 2012 => PH domain crystal with ligands) regarding Slm1 PH domain
These new citations have been included in lines 60-64.

v/ Line 75-6: Not all readers are familiar with the ATP analogs used to inhibit AS kinases.
We agree and have revised the manuscript to provide a brief explanation of the ATP analogs used to inhibit yck2-as1 kinase, along with a citation that explains development and use of the analogs.

vi/ Fig 2C: why is there much more Mss4-3xHA in lane 4 than in all others?? makes it a bit hard to conclude about a small shift, when loading not equal.
We agree that there was inconsistent protein loading or western blot transfer in the figure that made it harder to evaluate. To address this, we revised the figure (Fig 2D) to show an experimental replicate with more consistent protein detection. We also simplified the figure to focus on data showing that Yck1/2 can hyperphosphorylate Mss4. The previous version showed that Yck1/2 undergoes autophosphorylation, but we realized that was redundant with the following figure.

vii/ Sentences 118-120 are confusing.
We have edited these sentences to improve clarity.

viii/ The authors keep saying that “loss of Rts1 causes hyperphosphorylation of Mss4 but does not lead to full hyperphosphorylation” – I don´t understand what they mean. What is full hyperphosphorylation?
We agree that this is confusing and have edited the manuscript to clarify. By “full hyperphosphorylation” we meant that all Mss4 would be detected in the hyperphosphorylated form with no faster migrating unphosphorylated or partially phosphorylated forms.

ix/ It is unfortunate that the AS kinase appears to be a strong hypomorph. This limits the utilityof this approach.
Nearly all analog sensitive alleles are hypomorphs (e.g. PMID: 11014197), which must be considered when interpreting results. Nevertheless, they have proven to be powerful tools in a large number of studies. We have another paper that will soon be on bioRxiv that utilizes the new yck2-as1 allele to gain insight into growth-dependent signals that influence the

© 2024. Published by The Company of Biologists under the terms of the Creative Commons Attribution License (https://creativecommons.org/licenses/by/4.0/).

duration and extent of bud growth, and we are sure that others will find the new alleles useful.

x/ many of the protein shifts are very subtle and the lack of repetitions makes their interpretation tenuous at best.
All of the experiments have been repeated for a minimum of three biological replicates that give consistent results, so there is not a lack of repetitions. We agree that the observed shift for in vitro phosphorylation of Ypk1 by Yck1/2 is subtle and we have therefore removed that figure panel, which does not change any of the key conclusions. We disagree that many of the shifts are subtle (see figures 2A, 2D, 3B, 3C, 4A, 4B, 4C, 5A, 6A, S1, S2).

xi/ Line 201: there is no figure 5D
This has been corrected.

In summary, this seems like a somewhat random collection of data, the majority of which are negative results. Although alternative hypotheses are presented, these generally haven't been followed up. The more positive/striking results have already been published by these authors some years ago (10.1016/j.cub.2017.11.069; 10.1534/genetics.118.301296). We cannot recommend publication of this manuscript.
We disagree that the majority of the results are negative results. Most of the findings show clear effects of inhibiting Yck1/2 and provide solid data that future researchers can use to develop and refine new models for the functions of casein kinase 1 homolog and regulation of components of the TORC2 network. In addition, the new experiments requested by the reviewers have greatly improved the manuscript and show that Yck1/2 influence less well-defined aspects of the TORC2 network. Finally, we report new tools (the yck2-as1 allele and an anti-Yck1/2 antibody) that will be useful to the field.

Reviewer 2 Advance Summary and Potential Significance to Field...

This paper examines the role of the budding yeast casein kinase I proteins Yck1 and Yck2 in regulating activation of TORC2-Akt signaling. The authors test a proposed pathway that involves Yck1/2 phosphorylating PI(4,5)P2 kinase Mss4, leading to recruitment of Slm1/2 proteins and TORC2-Akt activation. Yck1 and Yck2 are functionally redundant so that yck1 and yck2 mutants are viable, but the double mutant yck1 yck2 is lethal. Previous work from another group showed that conditional inactivation of yck1Δ yck2-ts mutant prevents phosphorylation of Mss4, leading to this proposed pathway. In the current study, the authors generate an analog-sensitive yck2-as mutant with the goal of testing the pathway more precisely. The yck2-as mutation is a constitutive partial loss-of-function, so combined with yck1Δ provides a partial loss of Yck1/2 function. In this mutant, the authors test phosphorylation of various proteins in the pathway using western blot of whole cell extracts, combined with several in vitro assays using purified proteins. Their results provide a number of new insights for the pathway including: Yck1 might directly phosphorylate Mss4, Yck1/2 activity impacts TORC2 phosphorylation, Yck1/2 and PP2A phosphatase regulate each other's phosphorylation (though probably indirectly), and combined mutations in this pathway impact cell size and viability. The experiments are generally performed well, and the data interpreted appropriately. Each experiment in set up to test a simple prediction for the proposed linear pathway. The results will be helpful for researchers in the yeast cell growth field and are likely to help TORC2-Akt researchers in other experimental systems. The limitation of the study is that the results do not clarify aspects of the pathway. This signaling network clearly involves extensive feedback and likely has other components that are currently unknown. The result is that each finding does not fit into a larger conclusion. The authors are not to blame for the complex network, but it does limit the larger impact of the work. Most of the results lack an underlying mechanism and only tell us that mutant X somehow alters the phosphorylation of protein Y. The ability of the field to understand a complex signaling network requires somebody to test each of these interactions, as the authors have done here. Therefore, the work is important for the field to know, but immediate impact and conclusions are somewhat limited.

Reviewer 2 Comments for the Author...

© 2024. Published by The Company of Biologists under the terms of the Creative Commons Attribution License (https://creativecommons.org/licenses/by/4.0/).

Specific comments:
1. It would be helpful for the authors to test the impact of 3-MOB-PP1 drug on yck1Δ cells, at least for a couple of their assays/results. They need to control for effects of the drug that are independent of the yck2-as1 mutation.
We have performed the requested control experiments using yck1Δ, yck2Δ and cells treated with 3-MOB-PP1. These results are now included in the revised manuscript in Supplemental Figure 1 and 2A and the results are discussed in the text.

2. In Figure 2C, the loading does not appear equal for the final 2 lanes in the gel. It was hard for me to assess the gel shift for Mss4-3xHA given the difference in intensity between the bands.
We agree that there is inconsistent protein loading or western blot transfer in the figure that made it harder to evaluate. To address this, we revised the figure (Fig 2D) to show an experimental replicate with more consistent protein detection. We also simplified the figure to focus on data showing that Yck1/2 can hyperphosphorylate Mss4. The previous version showed that Yck1/2 undergoes autophosphorylation, but we realized that was redundant with the following figure.

3. In Figure 4A, the authors conclude that prolonged incubation with inhibitor alters Ypk1 phosphorylation pattern. I do not see any effect supporting the conclusion.
We have edited the manuscript to better explain the effect, which can be seen most strongly in the bottom panel at 60 minutes.

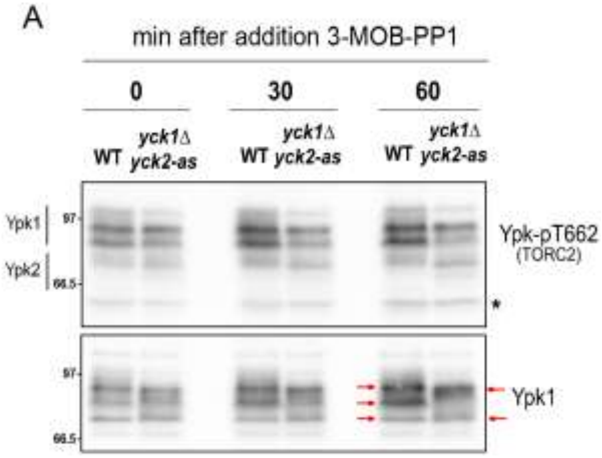

4. Minor: on line 170, the text states Ypk1/2 but I think they mean Yck1/2.
Thanks for catching this error, which has been corrected.

5. For Figure 5A, it would be helpful to see this experiment performed without inhibitor. Given the minimal effect of inhibitor addition in most experiments, I was confused why it was included here.
We carried out new experiments with and without inhibitor and have included a revised Figure 5A that includes the new data. The effect of single deletions *yck1Δ* and *yck2Δ* on Rts1 is also included in a new supplemental figure 2A.

6. Based on the model and background information, I am confused why loss of membrane localization does not impact Ypk-pY662 levels in Figure 6. Is it known where Ypk proteins localize in akr1Δ cells shifted to poor carbon? If not, the authors could perform this experiment to clarify their results in Figure 6.
We agree that it was surprising to find that inhibition of Yck1/2 and akr1Δ do not show stronger effects on TORC2-dependent phosphorylation of Ypk1/2. However, new Mss4 localization data show the surprising result that inhibition of Yck1/2 leads to increased localization of Mss4 to the cell periphery, which based on previous models would drive an increase in TORC2 signaling to Ypk1/2. Together, the data indicate that the regulation and function of Mss4 phosphorylation is more complex than previously imagined, and that Yck1/2

© 2024. Published by The Company of Biologists under the terms of the Creative Commons Attribution License (https://creativecommons.org/licenses/by/4.0/).

likely influence poorly understood aspects of the TORC2 network that may not be directly related to TORC2-dependent phosphorylation of Ypk1/2. We have revised the manuscript to highlight these new results and how they inform models for TORC2 function.

7. Line 242: The text states “these results are consistent with a model in which PP2A dephosphorylates and activates Ypk1/2.” I think they mean “inactivates”? Isn’t the idea that phosphorylation activates Mss4?
This was an error, and we meant to refer to Yck1/2. To clarify, we have revised the sentence and referred to the correct kinases.

8. It would be helpful to know where Mss4 localizes in yck1/2 mutants, based on changes identified in Mss4 phosphorylation.
We carried out new experiments to compare the localization of Mss4 in wild type and *yck-as* cells, which are shown in a new Figure 2B. The data indicate that loss of Yck1/2 causes increased localization of Mss4 to the plasma membrane, as well as preferential localization of Mss4 to the growing bud. This was surprising because previous work suggested that localization of Mss4 to the plasma membrane is correlated with phosphorylation of Mss4. A model that could explain the data is that there are distinct sets of phosphorylation sites on Mss4 that have distinct functions and undergo differential regulation. Overall, the data show that the functions and regulation of Mss4 phosphorylation are more complex than previously imagined.

The new data and other data in the manuscript indicate that Yck1/2 influence multiple components of the TORC2 network, but do not strongly influence phosphorylation of Ypk1/2 by TORC2, which is the best understood signaling axis in the TORC2 network. Inhibition of Yck1/2 therefore helps reveal aspects of the TORC2 network that are much less well-understood, and our hope is that these new data will provide important clues that lead to new insight into the TORC2 network. We have modified the abstract and text to incorporate discussion of these new data.

Second decision letter

MS ID#: JOCES/2024/262036

MS TITLE: Casein kinase 1 controls components of a TORC2 signaling network in budding yeast

AUTHORS: Rafael Lucena, Akshi Jasani, Steph Anastasia, Douglas Kellogg, and Maria Alcaide Gavilan

Your revised manuscript has now been assessed by the referees. The referees acknowledge that the work is carefully done and provocative. While the referees are less convinced about the potential impact of the work given the number of open questions, I have decided that it is in the best interest of the field to accept the work for publication in the Journal of Cell Science, pending standard publication integrity checks. I would ask that you consider the suggestions outlined in the referee reports as you prepare your final version for publication, as addressing these remaining concerns of the reviewers with additional editing would enhance the positive reception of your work. The reports are appended below.

Reviewer 1

Advance summary and potential significance to field

I remain of the opinion that the work in this manuscript is too preliminary for publication in its present form. The authors have published technically sound results, but a clear understanding of the signaling pathways do not emerge and the impact of this work will be correspondingly low.

Comments for the author

© 2024. Published by The Company of Biologists under the terms of the Creative Commons Attribution License (https://creativecommons.org/licenses/by/4.0/).

125 To further test whether Yck1/2 influence Mss4 function, we determined whether 126 normal localization of Mss4 is dependent upon Yck1/2 activity. Wild type and yck2-as1
127 yck1Δ cells were treated with 3-MOB-PP1 for 30 min and localization of Mss4-GFP was
128 analyzed. (Figure 2B, C). In wild type cells, Mss4-GFP was localized primarily to the
129 periphery of both mother cells and daughter buds, as previously reported (Audhya, 2003;
130 Lucena et al., 2018). In yck2-as1 yck1Δ cells, there was increased localization of Mss4 to 131 the periphery, as well as preferential localization of Mss4 to the growing daughter bud.
132 This was surprising because previous work suggested that localization of Mss4 to the 133 plasma membrane is correlated with phosphorylation of Mss4 (Audhya, 2003; Lucena et 134 al., 2018).
Localization appears to be unaffected by addition of analog: Might this just be an indirect consequence of low Yck1/2 activity?

Mss4 to the growing bud. Overall, the data show that the function 143 and regulation of Mss4
144 phosphorylation is more complex than previously imagined.
Unless clarity is brought to this complexity, I do not see this work fit for publication in this journal.

165 Both underwent extensive autophosphorylation in vitro that could be detected as an
166 electrophoretic mobility shift (Figure 3B and 3C). Furthermore, purified PP2ARts1 was able
167 to oppose autophosphorylation of Yck1 and Yck2 in vitro (Figure 3B and 3C).
Autophosphorylation was still evident even in the presence of PP2ARts1, indeed there is a quantative shift in protein mobility – do the authors propose that PP2ARts1 affects the ability of Yck1 to autophosphorylate some sites but not others? Would it not make more sense that PP2ARts1 dephosphorylates some but not all autophosphorylation sites in Yck1?
319 PP2ARts1 purified from yeast was not able to dephosphorylate Mss4 in vitro which
320 suggests that it acts indirectly.
Figure 3 would argue otherwise

179 Yck2 phosphorylation. These observations suggest that forms of PP2A that include Cdc55 or
180 Rts1 cannot be solely responsible for Yck2 dephosphorylation; however, it is possible that
181 PP2ARts1 and PP2ACdc55 work redundantly to mediate PP2A-dependent phosphorylation of Yck2.
182 It is also possible that another form of PP2A is responsible for directly controlling Yck2
183 phosphorylation. For example, previous studies have shown that PP2A catalytic subunits can
184 also associate with TAP42, which is thought to be regulated by nutrient-
dependent signals (Di
185 Como and Arndt, 1996).
Why not test these models?
198 other than T662 that cause changes in electrophoretic mobility. We found that T662
199 phosphorylation was modestly reduced in yck2-as1 yck1Δ cells in the absence of inhibitor
200 (Figure 4A). Addition of 3-MOB-PP1 did not cause a substantial further reduction in pT662
201 levels (Figure 4A). This result is similar to the result obtained for Mss4 where yck2-as1 caused
202 a loss of phosphorylation of Mss4 in the absence of inhibitor and addition of inhibitor caused
203 only minimal further effects.
Test in rts1D background?

227 dependent upon Yck1/2. We found that Fpk1 phosphorylation that can be detected via
228 electrophoretic mobility shifts was strongly reduced in yck2-as1 yck1Δ
cells, consistent with the
229 possibility that Yck1/2 influence Ypk1 phosphorylation via Fpk1/2 (Figure 4C).
Or due to indirect consequence of chronic low Yck1/2 activity

230 To further investigate how Yck1/2 influence Ypk1/2, we analyzed localization of
231 Ypk1-GFP in yck2-as yck1Δ cells (Figure 4D). We found that a reduction in Yck1/2 activity
232 caused increased recruitment of Ypk1/2 to punctate structures at the cell periphery,
233 which suggests that Yck1/2-dependent phosphorylation events influence localization of
234 Ypk1/2. The identity of the punctate structures is unknown. The fact that reduced activity
235 of Yck1/2 does not cause an increase in TORC2 signaling (Figures 4A and 4B)
Indicates
236 that the structures are unlikely sites of TORC2 signaling. The structures do not appear
237 to correlate with the previously reported sites of Fpk1/2 localization at endosomes.

© 2024. Published by The Company of Biologists under the terms of the Creative Commons Attribution License (https://creativecommons.org/licenses/by/4.0/).

238 Additional experiments will be needed to identify and characterize the sites of increased 239 Ypk1 localization in yck2-as yck1Δ cells.
Again, or an artifact of chronically low Yck1/2 activity.

Does expression of hyperactive Ypk1D239A suppress phenotypes associated with akr1D or yck1/2 mutants?

Reviewer 2

Advance summary and potential significance to field

The authors have revised the text to address most reviewer comments. They have also added several new experimental results. The revised paper is stronger than the initial submission and represents a nice contribution to the field. The main limitation remains its overall impact because the various signaling results do not fit coherently into a cohesive network or pathway. However, the authors' results provide new information about this network/pathway, and these results can be built upon by future studies.

Comments for the author

Below are several comments that could be addressed to strengthen the paper:
1. Figure 2B: This quantification should be performed on single focal plane images, instead of maximum intensity projections. If the authors prefer to use a projection of some kind, I suggest a sum projection of several middle focal planes. Maximum intensity projections are typically not appropriate for quantification, and the signal from top/bottom focal planes distracts from the ability to monitor changes in the cortical levels from middle focal planes. It would also be interesting to perform this experiment in rts1Δ cells, but looking at this mutant does not seem required for publication.

2. Line 200: change "did not cause a substantial further reduction" to "did not cause any reduction."

3. Lines 206-208 and Figure 4A: I do not agree with the authors' conclusion. There is no change at 30 minutes (which is enough time for the other results in the paper), and I am not convinced that there is a change at 60 minutes. I consider their conclusion to be an overinterpretation of the data.

4. Line 243: "Phosphorylation of these sites is influenced by the Fpk1/2 kinases…" What is the evidence for this statement? I don't remember it being tested.

5. Line 244: "…Yck1/2 appear to strongly influence regulation of Fpk1…" I would suggest removing "strongly" from this sentence, as the single result supporting this conclusion is modest (Figure 4C).

6. Lines 307-308: "…which indicates that PP2A-Rts1 cannot be ther only phosphatase that works on Mss4."
It seems entirely possible that not all Mss4 is subject to phosphorylation by Yck or other kinases. In this scenario, PP2A-Rts1 could still be the only phosphatase to work on Mss4.

7. It would be helpful for the authors to provide a cartoon network of these proteins and their interactions (arrows etc...). It is ok if there are a few question marks due to unknown steps, but a network summary would be helpful for keeping track of the various proteins and their known interactions.

© 2024. Published by The Company of Biologists under the terms of the Creative Commons Attribution License (https://creativecommons.org/licenses/by/4.0/).
